# Comparison of human poly-*N*-acetyl-lactosamine synthase structure with GT-A fold glycosyltransferases supports a modular assembly of catalytic subsites

**DOI:** 10.1074/jbc.RA120.015305

**Published:** 2020-12-03

**Authors:** Renuka Kadirvelraj, Jeong-Yeh Yang, Hyun W. Kim, Justin H. Sanders, Kelley W. Moremen, Zachary A. Wood

**Affiliations:** 1 Department of Biochemistry & Molecular Biology, University of Georgia, Athens, Georgia, USA; 2 Complex Carbohydrate Research Center, University of Georgia, Athens, Georgia, USA

**Keywords:** glycosyltransferase, substrate recognition, enzyme mechanism, SEC-MALS, size-exclusion chromatography coupled with multiangle light scattering

## Abstract

Poly-*N*-acetyl-lactosamine (poly-LacNAc) structures are composed of repeating [-Galβ(1,4)-GlcNAcβ(1,3)-]_n_ glycan extensions. They are found on both *N*- and *O*-glycoproteins and glycolipids and play an important role in development, immune function, and human disease. The majority of mammalian poly-LacNAc is synthesized by the alternating iterative action of β1,3-*N*-acetylglucosaminyltransferase 2 (B3GNT2) and β1,4-galactosyltransferases. B3GNT2 is in the largest mammalian glycosyltransferase family, GT31, but little is known about the structure, substrate recognition, or catalysis by family members. Here we report the structures of human B3GNT2 in complex with UDP:Mg^2+^ and in complex with both UDP:Mg^2+^ and a glycan acceptor, lacto-*N*-neotetraose. The B3GNT2 structure conserves the GT-A fold and the *DxD* motif that coordinates a Mg^2+^ ion for binding the UDP-GlcNAc sugar donor. The acceptor complex shows interactions with only the terminal Galβ(1,4)-GlcNAcβ(1,3)- disaccharide unit, which likely explains the specificity for both N- and O-glycan acceptors. Modeling of the UDP-GlcNAc donor supports a direct displacement inverting catalytic mechanism. Comparative structural analysis indicates that nucleotide sugar donors for GT-A fold glycosyltransferases bind in similar positions and conformations without conserving interacting residues, even for enzymes that use the same donor substrate. In contrast, the B3GNT2 acceptor binding site is consistent with prior models suggesting that the evolution of acceptor specificity involves loops inserted into the stable GT-A fold. These observations support the hypothesis that GT-A fold glycosyltransferases employ coevolving donor, acceptor, and catalytic subsite modules as templates to achieve the complex diversity of glycan linkages in biological systems.

Glycans attached to protein Asn (*N*-linked glycoproteins) and Ser/Thr (*O*-linked glycoproteins) side chains and glycolipids are commonly extended by the addition of disaccharide repeats ([-Galβ(1,4)-GlcNAcβ(1,3)-]_n_) on one or more terminal branches of the respective structures (Fig. 1) (1). These glycan extensions, termed poly-*N*-acetyl-lactosamine (poly-LacNAc) structures, can act as backbone polymers for additional glycan branching (I-antigen structures (2, 3)), modification (*e.g.*, sulfation to form keratan sulfate (4)), or addition of unique terminal capping structures (*e.g.*, ABO (5), Lewis blood group (6), or HNK-1 (7) antigen structures). Poly-LacNAc extensions can be found on all classes of complex multiantennary *N*- and *O*-glycan structures as well as glycolipids and are prevalent on highly branched glycan products (Fig. 1). The linear poly-LacNAc polymer backbone can be recognized as a ligand for endogenous lectins (galectins) in a variety of cellular contexts (8, 9), and the various terminal capping structures play roles in diverse biochemical processes (*e.g.*, the role of sialyl Lewis X structures in targeting lymphocytes to sites of inflammation (10, 11)). Altered extension of poly-LacNAc structures has also been described in cancer cells suggesting that these structures may regulate cell adhesion processes during cancer malignancy (4, 12, 13, 14, 15, 16, 17).

**Figure 1:**
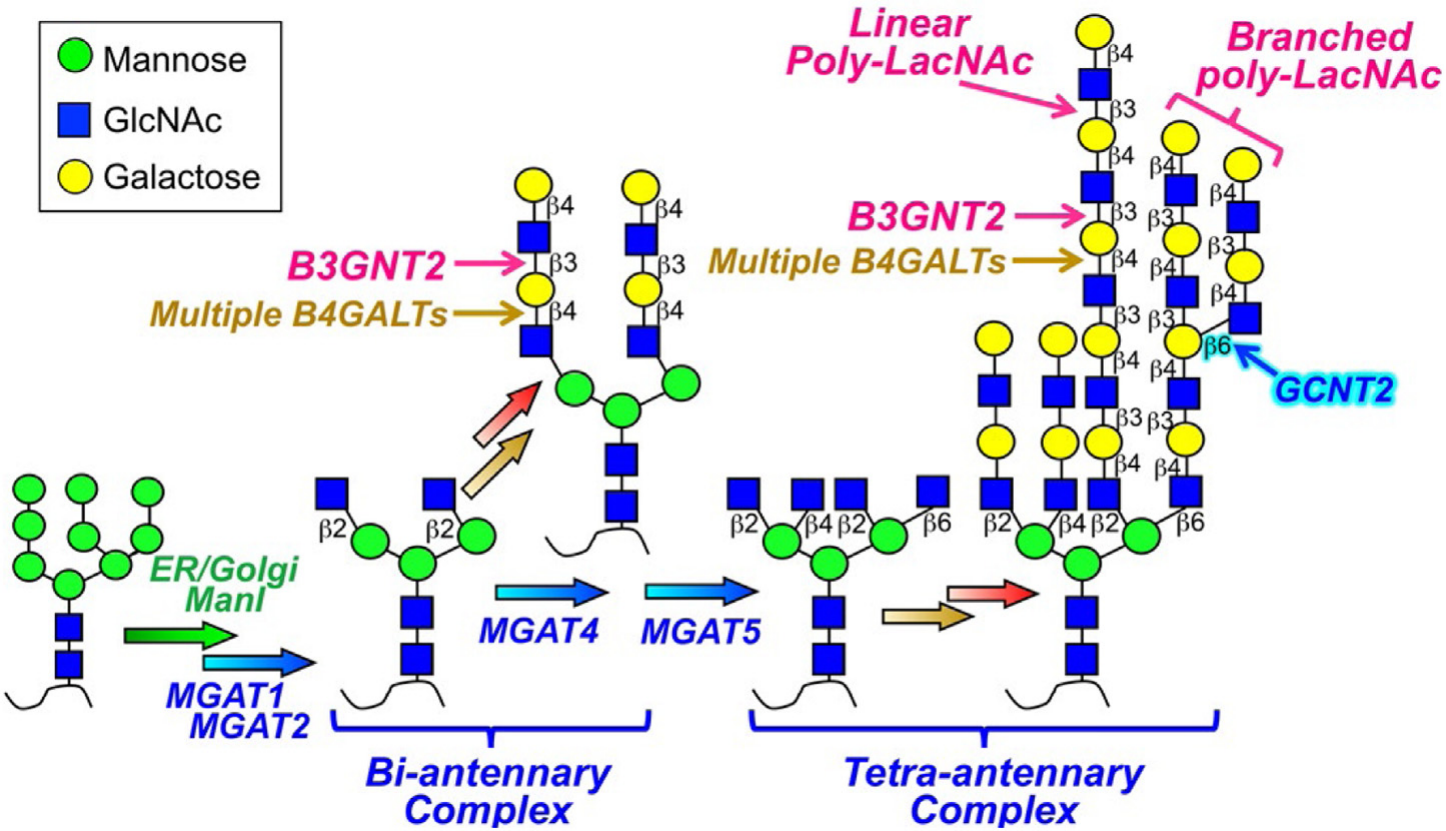
**The synthesis and extension of polylactosamine.** Polylactosamine structures are generated on the terminal branches of multiantennary complex N-glycans by the alternating actions of B4GALTs and B3GNTs. The I-branching enzyme GCNT2 can add β1,6-branches (highlighted in *cyan*), which are then subject to polylactosamine elongation and terminal modifications.

The synthesis of poly-LacNAc structures requires the iterative action of two enzymes: β1,4-Gal transferases (B4GALTs) and β1,3-GlcNAc transferases (B3GNTs) (Fig. 1) (18, 19, 20, 21). B4GALTs are classified into family GT7 in the CAZy database (22) and are broadly expressed (23) with several isoforms being capable of generating both single LacNAc units and extended poly-LacNAc polymers. There are eight mammalian B3GNTs belonging to family GT31, with each exhibiting a different expression pattern in animal tissues (24). These eight isozymes appear to act through a cation(metal)-dependent inverting catalytic mechanism, each with a distinct specificity for *N*-glycan, *O*-glycan, or glycolipid poly-LacNAc extension (25, 26). Among these, B3GNT2 is the most abundant and ubiquitously expressed B3GNT isoform (30) and displays the greatest *in vitro* activity for *N*-glycan and *O*-glycan poly-LacNAc extension (10, 19, 25). These observations led to the conclusion that B3GNT2 was the principal poly-LacNAc synthase in mammalian organisms. This hypothesis is supported by the observation that *B3gnt2*-knockout mice display a significant reduction in polylactosamine structures (27), with defects in olfactory bulb innervation and glomerular formation (28), altered immune regulatory functions (11), and reduced reproductive rates (29).

B3GNT2 shows high activity toward glycan poly-LacNAc substrates containing variable numbers of LacNAc repeats, which suggests that the acceptor specificity is limited to the terminal LacNAc unit (10). Unfortunately, there are no crystal structures of an acceptor-bound β1,3-GlcNAc transferase to predict the substrate specificity determinants of B3GNT2. In fact, the only crystal structure of a β1,3-GlcNAc transferase is that of the distantly related GT31 enzyme, mouse manic fringe (Mfng), in complex with UDP (30).

To understand the structural basis of acceptor recognition and poly-LacNAc synthesis for human B3GNT2, we crystallized the catalytic domain as a donor analog complex (UDP:Mg^2+^) and a ternary complex with donor analog and acceptor (UDP-Mg^2+^:lacto-*N*-neotetraose). The structures revealed a GT-A fold enzyme containing a *DxD* motif for metal-dependent interactions with the donor substrate and an acceptor binding site that interacts with the terminal LacNAc unit of the acceptor. Structural comparisons with other GT-A fold inverting glycosyltransferases show that the binding site geometry for positioning the sugar donor is conserved despite sequence variability among the corresponding interacting residues. In contrast, the acceptor subsites were assembled from structural elements inserted into solvent-exposed loops of the core GT-A fold. Our structural analysis provides a framework for understanding the molecular basis for polylactosamine biosynthesis by B3GNT2 and how the evolution of GT-A fold enzymes makes use of modular donor and acceptor templates for the assembly of diverse glycan linkages in biological systems.

## Results

### B3GNT2 expression, purification, structure determination, and dimerization

**Figure 2:**
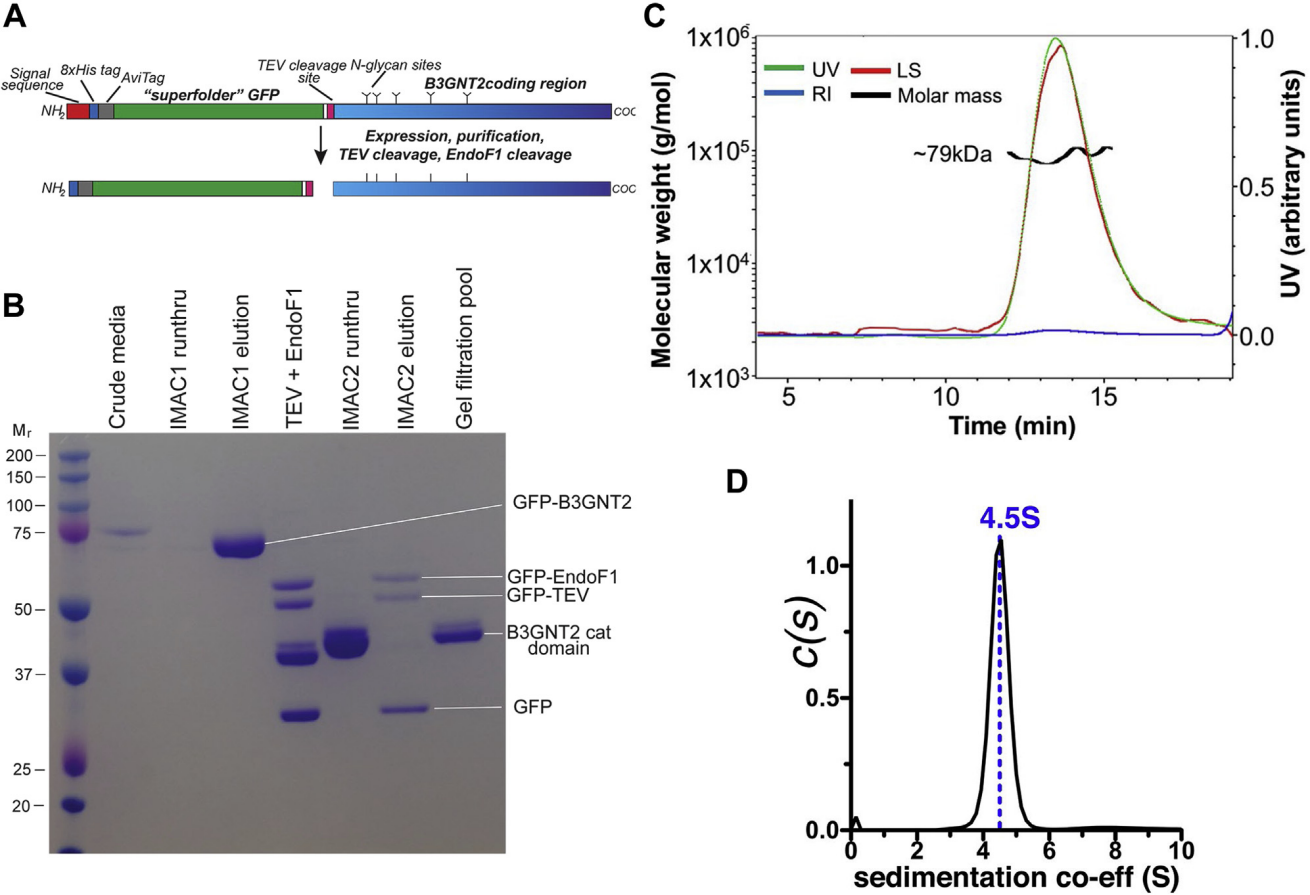
**Expression, purification, and dimerization of the catalytic domain of human B3GNT2.***A*, diagrammatic representation of the recombinant B3GNT2 fusion protein coding region is shown. This fusion protein has an NH_2_-terminal signal sequence followed by an 8xHis tag, AviTag, superfolder GFP, TEV protease cleavage site, and the catalytic domain of B3GNT2 containing five N-glycan consensus sequons sites at N79, N89, N127, N173, and N219. *B*, expression of the recombinant product in HEK293S (GnTI-) cells resulted in secretion of the fusion protein into the culture medium (Crude media), and subsequent Ni^2+^-NTA purification yielded a highly enriched enzyme preparation (IMAC1 elution). Cleavage of the enzyme with TEV protease and EndoF1 resulted in removal of the tag sequences and glycans, leaving only a single GlcNAc residue attached to each Asn side chain (TEV + EndoF1, B3GNT2 cat domain). Ni^2+^-NTA chromatography separated the unbound B3GNT2 catalytic domain (IMAC2 run-thru) from the bound tag sequences, TEV protease, and EndoF1 (IMAC2 elution), as the latter were all His-tagged. The enzyme was further purified over Superdex-75 (Gel filtration pool). *C*, The purified B3GNT2 catalytic domain following cleavage with TEV and EndoF1 was further characterized by size exclusion–multiangle light scattering (SEC-MALS). A_280_ is shown by the *green line*, refractive index in *blue*, light scattering in *red*, and calculated molar mass in *black*. The molecular mass derived from SEC-MALS analysis (∼79 kDa) is in close agreement with a dimeric form of the B3GNT2 catalytic domain monomer following cleavage with TEV and EndoF1 (∼42 kDa). *D*, the c(s) distribution for the sedimentation of B3GNT2 in 250 mM NaCl and 20 mM HEPES, pH 7.4, shows a peak at a sedimentation coefficient of 4.5 S indicating dimer (calculated value is 4.9 S).

**Figure 3:**
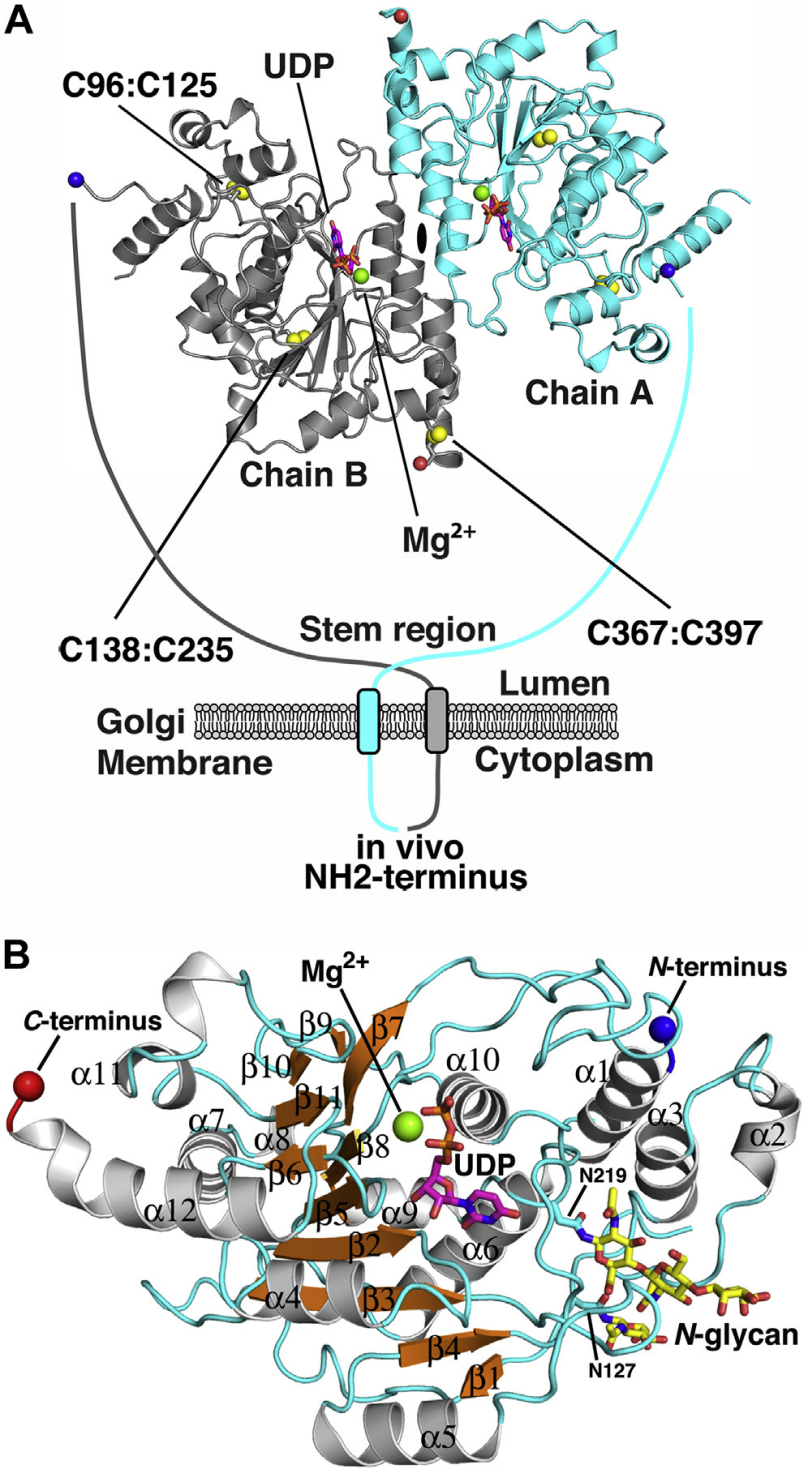
**The donor nucleotide sugar binding site in B3GNT2.***A*, The structure of SeMet-B3GNT2:UDP:Mg^2+^ showing the dimer in the asymmetric unit (*cyan* and *gray* cartoons). The UDP (*magenta*, *stick mode*) and Mg^2+^ (*green sphere*) in the active site and the disulfide bonds (*yellow spheres*), the N-terminus of the structure (*blue spheres*), and C-terminus (*red spheres*) are shown. The proposed membrane bound form of the SeMet-B3GNT2:UDP:Mg^2+^ dimer is depicted with a diagrammatic representation of the “stem region” (residues 29–51, *cyan* and *gray arcs*) and NH_2_-terminal transmembrane anchor (residues 8–28, *cyan* and *grayrectangles*) for the full-length enzyme found *in vivo*. *B*, The structure of the SeMetB3GNT2:UDP:Mg^2+^monomer in cartoon mode with secondary structural elements colored as follows: helices (*pale gray*), sheets (*orange*), and loops (*cyan*). The secondary structure elements are numbered sequentially. The active site UDP, the Mg^2+^ ion, the N-and C- termini are shown and colored as in panel A. The N-glycans attached to N127 and N219 are also shown and colored *yellow*.

The catalytic domain of B3GNT2 (residues 34–397) was expressed as a secreted fusion protein in HEK293S (GnTI-) cells in the presence and absence of metabolic labeling with selenomethionine (SeMet) and purified using previously described workflows (Fig. 2, *A–B*) (31, 32). The crystal structure of SeMet-B3GNT2 in complex with the donor analog UDP and Mg^2+^ (SeMet-B3GNT2:UDP:Mg^2+^) was solved at 1.55 Å resolution using single-wavelength anomalous diffraction (Table 1). Two molecules were found in the asymmetric unit of the SeMet-B3GNT2:UDP:Mg^2+^ structure that were arranged as a dimer with a ∼924 Å^2^ interface formed between helices α4, α5 and α12 from each chain (Fig. 3*A*, Fig. S1). Unlabeled B3GNT2:UDP:Mg^2+^ crystallized in a different space group and was solved at 2.04 Å resolution (Table 1). Despite differences in crystal packing, the SeMet-B3GNT2:UDP:Mg^2+^ and B3GNT2:UDP:Mg^2+^ structures are essentially the same, with the latter revealing the same dimeric complex formed by crystallographic symmetry. The dimer was also observed by size exclusion–multiangle light scattering (SEC-MALS), which revealed a single broad peak eluting with a predicted molecular mass of ∼79 kDa (Fig. 2*C*). Sedimentation velocity analysis showed a single 4.5 S species, which is slightly less than the 4.9 S value predicted using the crystallographic dimer (Fig. 2*D*). The slower sedimentation likely results from two disordered segments (residues 34–56 and 72–90) that were not modeled in the crystal structure, but would increase the frictional coefficient of the protein. PISA analysis predicts that the dimer interface we observe in both crystal forms of B3GNT2 is stable with a favorable solvation-free energy gain (Δ^i^G) of -17.5 kcal mol^-1^ and a *p*-value of 0.007 (Table S1), which indicates that the surface is interaction-specific (an authentic interface is expected to have a *p*-value <0.5 (33)).

**Table 1:** Data collection and refinement statistics

Data collection	SeMet-B3GNT2:UDP-Mg^2+^	B3GNT2:UDP-Mg^2+^	B3GNT2:UDP-Mg^2+^:LNnT
Wavelength (Å)	0.9500	1.0000	1.0000
Space group	P 2_1_ 2_1_ 2_1_	P 2_1_	P 2_1_ 2_1_ 2_1_
Unit cell dimensions a, b, c (Å) and α,β,γ (°)	48.73, 110.92, 147.40 90.0, 90.0, 90.0	67.21, 79.81, 157.79 90.0, 97.89, 90.0	48.19, 109.28, 147.55 90.0, 90.0, 90.0
Molecules in asymmetric unit	2	4	2
Completeness (%)	99 (93)^a^	100 (99)^a^	100 (96.4)^a^
Total reflections	1700923 (108816)	790241 (56,301)	947780 (46,831)
Unique reflections	224051 (15,467)	105155 (7723)	67,358 (4731)
Redundancy	7.6 (7.04)	7.5 (7.3)	14.1 (9.9)
*I*/σ(*I*)	12.97 (0.89)	13.9 (0.99)	24.40 (1.01)
R-meas^b^ (%)	0.093 (2.75)	0.095 (2.44)	0.063 (2.27)
*CC*_1/2_^c^ (%)	0.999 (0.436)	0.999 (0.511)	0.999 (0.449)
Refinement			
Resolution (Å) and highest resolution shells	1.55 (1.59–1.55)	2.04 (2.09–2.04)	1.85 (1.90–1.85)
R_*work*_/R_*free*_	0.177/0.198	0.218/0.232	0.184/0.214
No. atoms Protein/Ligand/Water	5390/208/478	10,517/340/289	5317/290/241
*B*-factor (Å^2^) Protein/Ligand/Water	30.6/34.1/40.1	59.3/52.5/50.7	49.1/55.9/48.3
Wilson *B*-factor (Å^2^)	22.9	42.3	37.0
Stereochemical Ideality			
Bond lengths (Å^2^)	0.015	0.004	0.016
Bond angles (°)	1.43	0.7	1.4
Ramachandran favored (%)	98	98.2	96.7
Ramachandran allowed (%)	2	1.8	3.3
Ramachandran outliers (%)	0	0	0
SAD Phasing statistics			
Heavy atom sites in asymmetric unit	10		
Figure of merit	0.34		
PDB code	6WMM	6WMN	6WMO

a Values in parentheses are for highest-resolution shell.

b R_meas_ is the redundancy independent merging R-factor of Diederichs and Karplus (77).

c CC_1/2_ is the percentage of correlation between intensities from random half-data sets (78).

B3GNT2 also has five predicted N-glycosylation sites at asparagine residues 79, 89, 127, 173, and 219 (Fig. S1) (34). There is unambiguous electron density for the two GlcNAc residues of a trisaccharide glycan (Manβ1,4-GlcNAcβ1,4-GlcNAcβ-) attached to Nδ of Asn219 and weaker density for the solvent-exposed β-mannose (Fig. S2*A*). The remaining mannose residues are disordered or missing in the structure. The two GlcNAc residues are buried in a cleft that likely prevented deglycosylation of the glycan structure during purification from the HEK293S (GnTI-) host cells (Fig. S2*B*). The cleft is formed between helix α6 from the GT-A fold and an NH_2_-terminal insertion into the fold (residues 54:137) (Figs. S1 and S2*B*). The extensive packing interactions between the cleft residues and the glycan suggest that this glycosylation site is important for protein folding or stability and has been previously identified as a conserved glycosylation site in B3GNT and B3GALT family members in GT31 (35). For the remaining predicted sites, Asn127 and Asn173 display weak electron density for a single GlcNAc residue, and Asn79 and Asn89 are part of the disordered NH_2_-terminus in the SeMet-B3GNT2:UDP:Mg^2+^ structure.

### UDP:Mg^2+^ interactions in the donor subsite

**Figure 4:**
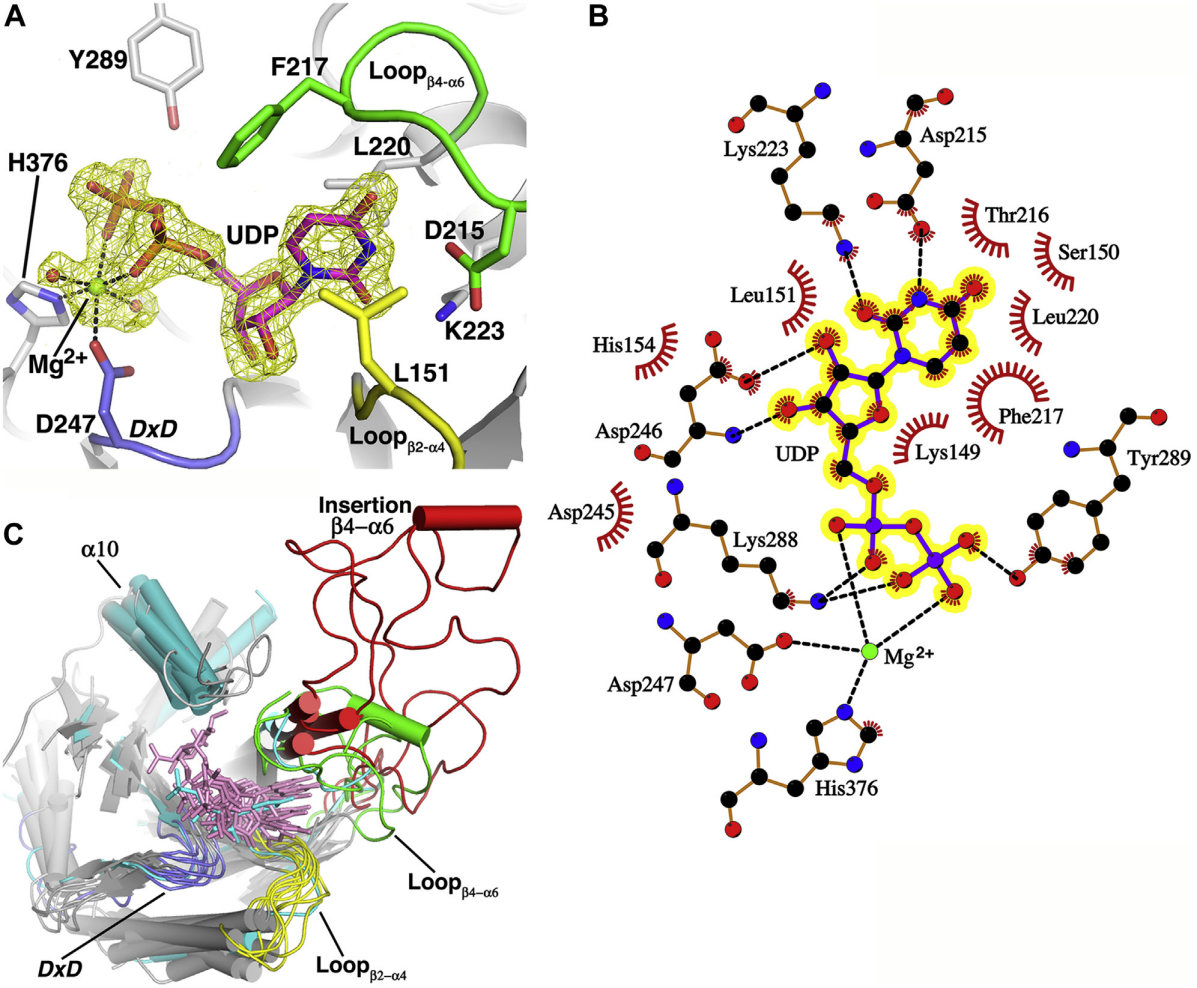
*A*, Difference density map (F_o_–F_c_, *yellow mesh*) for the UDP and Mg^2+^ in the B3GNT2 nucleotide sugar binding pocket (*pale gray cartoon*) calculated at 1.55 Å and contoured at 3.5 σ. The map was calculated subsequent to the structure solution and an initial round of restrained refinement but prior to the modeling of the ligands. The UDP and Mg^2+^ are from the final refined coordinates of SeMet-B3GNT2:UDP:Mg^2+^ and colored as in Figure 3*A*. Amino acid side chains (*stick mode*) and structural elements Loop_β2-α4_ (*yellow*), Loop_β4-α6_ (*green*), and Loop_β5-β6_ (*DxD* motif, *slate blue*) that interact with the UDP are shown. The octahedral coordination geometry of the metal ion involving the nucleotide diphosphate, side chains of H376 and D247 (last residue in the *DxD* motif), and solvent molecules (*red spheres*) is indicated with *black dashes*. *B*, Ligplot (79) representation of the B3GNT2 active site (*orange, ball* and *stick*) showing packing interactions (*red, feathered lines*) and hydrogen bonds (*black, dashed lines*) of the UDP (*yellow highlight*) and Mg^2+^ ion (*green*). *C*, The common active site architecture of GT-A fold glycosyltransferases (*pale gray, cartoon*) results in conformational similarity of bound nucleotide sugar donors and donor analogs (*pink sticks*). The donor binding pocket is shown with the structural elements Loop_β2-α4_, Loop_β4-α6_, and Loop_β5-β6_ (*DxD* motif) that interact with the nucleotide colored as in Panel A. For some GT-A fold enzymes, Loop_β4-α6_ can form extended regions (*red*) (*e.g.*, GT6 (GGTA1), GT13 (MGAT1 and POMGNT1), and GT16 (MGAT2)). Helix α10, the location of the catalytic base in inverting GT-A fold GTs, is colored *teal*. The representative subset of GT-A fold structures from CAZy GT families 2, 6, 7, 13, 14, 16, 31, and 43 were aligned with B3GNT2 using the core GT-A fold (see Table S2).

The SeMet-B3GNT2:UDP:Mg^2+^ structure reveals a GT-A Rossmann-like fold (36) comprised of eight twisted β-strands and 12 α-helical segments (Fig. 3*B* and Fig. S1). The donor nucleotide sugar binds in a pocket located at the carboxy end of the GT-A fold β1 and β4 strands (secondary structure elements refer to the core GT-A fold nomenclature in Fig. S1 and Fig. 3*B*). There is unambiguous electron density for the Mg^2+^ and UDP bound in the pocket (Fig. 4*A*). One side of the pocket is formed from residues in the Loop_β4-α6_ and the N-terminus of the α3 helix that interact with the uracil base. Asp215 and Lys223 form hydrogen bonds with the uracil N3 and O2 atoms, respectively (Fig. 4, *A–B*). Residues Thr216, Phe217, and Leu220 form a complementary surface that cradles the uracil ring, which is in turn sandwiched by Leu151 in Loop_β2-α4_. The other side of the nucleotide sugar binding pocket is formed by Loop_β5-β6_, which contains the *DxD* motif (Asp245-Asp246-Asp247) (Fig. 4, *A–B*). The carboxylate of Asp245, the first residue in the *DxD* motif, is pointing into the active site where one would expect to find the donor sugar. The carboxylate and backbone amide of Asp246 form hydrogen bonds with the nucleotide ribosyl 2′ and 3′ hydroxyls, respectively. The last residue in the *DxD* motif, Asp247, acts as a ligand for coordinating the Mg^2+^ cation. His356 and two water molecules also interact with the metal. Two phosphate oxygens from the nucleotide diphosphate (one each from the α- and β-phosphate) act as the final two ligands for the octahedrally coordinated Mg^2+^.

### Comparison of donor interactions with other GT-A fold glycosyltransferases

We compared the donor substrate interactions in B3GNT2 to the broad collection of crystal structures from representative GT-A fold glycosyltransferases in CAZy. These transferases are highly divergent, with residues in the core GT-A fold sharing sequence identities of <17% (Table S2). Despite the low degree of sequence conservation, the core structural elements of the GT-A folds were conserved, with the largest differences arising from loop insertions into the GT-A fold core (37). This set of GT-A fold glycosyltransferases display donor substrate diversity (enzymes employing UDP-GlcNAc, UDP-GalNAc, UDP-Glc, UDP-GlcA, UDP-Xyl, or GDP-Man sugar donors) as well as varied catalytic mechanisms that include metal-dependent and metal-independent inverting and metal-dependent retaining enzymes (38) (Table S2). Despite the differences in both donor specificity and the reaction mechanism, all of these enzymes use a common active site architecture to bind their respective sugar donors in a conformation similar to that observed in B3GNT2 (Fig. 4*C*). While the nucleotide donor pocket is formed from the same structural elements (Loop_β2-α4,_ Loop_β4-α6_ and Loop_β5-β6_), almost none of the interacting residues are conserved. Even among enzymes that bind the same donor substrate, the complementary interactions with the nucleotide sugar donor are not conserved at the sequence level, but instead originate from similar amino acids in similar positions of the fold (Fig. S4*B*). The only significant sequence conservation in the donor binding site is found in the *DxD* motif of metal-dependent GT-A fold enzymes (37). The first residue in the *DxD* motif is either an Asp or Glu, which interacts with the hydroxyl groups of the donor sugar to position the substrate (37, 39). The “x” residue is usually an acidic residue (Asp or Glu) or a small aliphatic. In the case of an Asp or Glu, the interaction is similar to what we observe in B3GNT2 (Fig. 4*B* and Fig. S4*B*). However, if the “x” is a small aliphatic, the ribosyl 3′ hydroxyl forms hydrogen bonds with the backbone amide of the aliphatic and the carbonyl group of a residue in the beginning of Loop_β2-α4_ of the GT-A fold (Fig. 4*C*). The last residue in the *DxD* motif is either an Asp or a Glu that acts as a ligand for coordinating the Mg^2+^ or Mn^2+^ cation. The metal-independent inverting glycosyltransferases, such as core 2 β-1,6-N-acetylglucosaminyltransferase (Gcnt1), do not conserve the *DxD* motif and satisfy the diphosphate interactions with the basic residues such as Arg and Lys (40, 41). The most significant differences in forming the binding site of the donor sugar involve Loop_β4-α6_. In some enzyme families such as GT13 (MGAT2 and POMGNT1) and GT16 (MGAT1) enzymes, the α6 helix is extended and may form part of the glycan acceptor binding site (*e.g.*, MGAT2 (42)) (Fig. 4*C*).

### B3GNT2 acceptor binding site evolved from inserted structural elements

**Figure 5:**
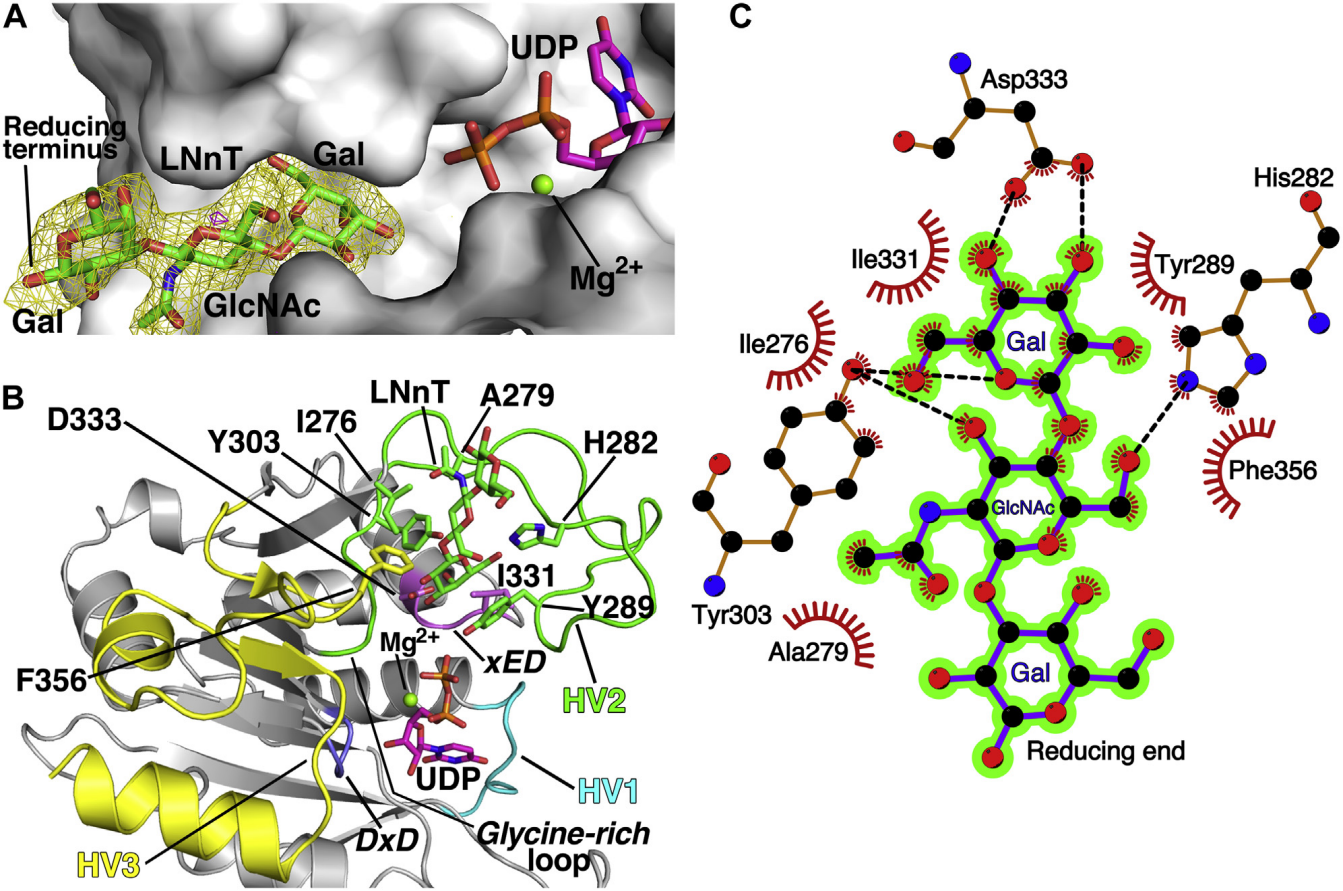
**The acceptor binding site in B3GNT2.***A*, Difference density map (F_o_-F_c_, *yellow mesh*) for the acceptor lacto-N-neo-tetraose (LNnT, *green*) in the B3GNT2 acceptor binding cleft (*pale gray surface*), calculated at 1.85 Å and contoured at 3.0 σ. The map was calculated after omitting LNnT from the refined coordinates of B3GNT2:UDP:Mg^2+^:LNnT and subjecting the model to simulated annealing. The Gal and GlcNAc units of LNnT (Galβ(1–4)-GlcNAcβ(1–3)-Galβ(1–4)-Glc), the UDP (*magenta*), and Mg^2+^ (*green sphere*) are shown. *B*, The B3GNT2 active site (*pale gray, cartoon*) with UDP, LNnT, and Mg^2+^ depicted and colored as in Panel A. Residue side chains (*sticks*) that are involved in acceptor interactions and the loop insertions in the core GT-A fold (HV1 (*cyan*), HV2 (*green*), and HV3 (*yellow*) are shown. The xED motif, the location of the catalytic base in inverting GT-A fold GTs, is colored *pink* and Loop_β5-β6_ (*DxD* motif) is colored *slate blue*. The side chain of the C-His involved in metal coordination is colored *orange* and the “Glycine-rich” loop is colored *red*. *C*, Ligplot (79) representation of the B3GNT2 active site showing the packing interactions (*red, feathered lines*) and hydrogen bonds (*black, dashed lines*) of the acceptor, LNnT (*purple, ball* and *stick*). The Gal and GlcNAc units of LNnT are highlighted in *green*.

The structure of B3GNT2 in complex with the tetrasaccharide acceptor analog, Lacto-N-neotetraose (Galβ(1–4)-GlcNAcβ(1–3)-Galβ(1–4)-Glc, LNnT), UDP, and Mg^2+^ (B3GNT2:UDP:Mg^2+^:LNnT) was solved to a resolution of 1.85 Å in the same crystal form as SeMet-B3GNT2:UDP:Mg^2+^ (Table 1). The nonreducing Gal and GlcNAc of LNnT are well ordered in a surface groove that extends into the donor sugar binding site (Fig. 5*A*). The distal Gal of LNnT is solvent exposed and weakly ordered, while the terminal Glc is disordered and could not be modeled (Fig. 5*A*). The B3GNT2 acceptor binding site is formed by two loop insertions into the GT-A fold core (Loop_β7-β8_ (residues 275–307) and Loop_α10-β11_ (residues 343–373)) as well as residues at the NH_2_-terminal end of α10 (Ile331-Asp332-Asp333). (Fig. S1 and Fig. 5*B*). The O3 and O4 sugar hydroxyls of the terminal Gal form hydrogen bonds with Asp333, while the O5 atom accepts a hydrogen bond from the hydroxyl of Tyr303 (Fig. 5*C*). The GlcNAc O3 donates a hydrogen to the Tyr303 hydroxyl while the O6 atom shares a hydrogen bond with His282. Finally, residues Ile276, Ala279, Tyr289, Ile331, and Phe356 form a complementary surface for van der Waals packing interactions with the acceptor (Fig. 5, *B–C*). Mutagenesis data support the roles of Phe356, Tyr303, Tyr289, and Ile276 in acceptor binding (with 19-, 9-, 230-, 423-, and 176-fold reductions in k_cat_/K_m_ for F356A, Y303A, Y289A, I276A mutants, respectively), while the contribution of His282 is relatively minor, with only a twofold reduction in k_cat_/K_m_ for H282A (Table S3).

The acceptor binding site in B3GNT2 was compared with a representative subset of GT-A fold glycosyltransferase crystal structures in complex with acceptor and donor analogs (Table S2). In contrast to the nucleotide sugar donor site, the acceptor binding sites are formed mostly by nonconserved structural elements inserted into loops in the core GT-A fold (Fig. S3). Prior evolutionary studies have identified four conserved landmark features among GT-A fold enzymes (37): the *DxD* motif for metal cation interactions, a “Glycine-rich” loop facing the acceptor and donor sugar site at the N-terminal end of the conserved β7 strand (β8 in B3GNT2, Fig. S1), an “xED” motif at the beginning of helix F (α10 in B3GNT2) harboring the catalytic base, and a “C-His” residue that coordinates with the metal ion. In addition, this prior work identified three positions in the Rossmann fold core where hypervariable loops (HV1, HV2, and HV3) were potentially inserted to provide acceptor binding subsites (37). We anticipated a similar positioning of key structural features in the B3GNT2 active site. Loop_β4-α6_ in B3GNT2 is equivalent to HV1 in the conserved GT-A common core, but has no significant insertions that contribute to acceptor interactions beyond the uracil base of the sugar donor as described above. The final four residues of HV2 form the “Glycine-rich” loop, as identified previously in Mfng (30). Residues of the “Glycine-rich” loop, the “xED” motif, and the “C-His” are also conserved in the B3GNT2 structure (Fig. S1) and play their respective anticipated roles based on the GT-A fold consensus structure.

In contrast, there are significant insertions into regions analogous to HV2 and HV3 in B3GNT2 that contain residues contributing to the acceptor binding site. HV2 loop in B3GNT2 (Loop_β7-β8_) is a long and convoluted 32 residue insertion that is devoid of secondary structure and contributes a majority of the acceptor binding site residues (five of seven interacting residues, Fig. 5*C*). Residues of the final HV3 loop (Gly251 to the C-terminus) also diverge from the GT-A fold common core following the final strand of the Rossmann fold β-sheet and form an extended 78 residue structure that includes Phe356 in the acceptor binding site and the His376 of the C-His motif that coordinates the Mg^2+^ ion. Additional acceptor interactions come from the conserved xED motif (Ile331 and Asp333 residues) (Fig. 5).

Comparison with other GT-A fold enzymes revealed numerous examples of acceptor interacting residues in the loop-α10 region associated with the xED motif and flanking residues (Fig. S4). However, these acceptor interactions generally involve primary sequence differences among the GT-A fold enzymes in this conserved loop-helix region rather than an insertion of a hypervariable loop sequence. Thus, the two final hypervariable loops and residues within the xED motif comprise the acceptor binding site for B3GNT2.

By comparison, the collection of GT-A fold enzymes depicted in the topology diagrams in Fig. S4 illustrates the range of positions for acceptor interactions that are achieved through insertions into HV1 (MGAT2), HV2 (B3GNT2, Gcnt1, GGTA1 and B4GALT1), and HV3 (B3GNT2, POMGNT1, MGAT2, Gcnt1, GGTA1, and B4GALT1) as well as sequences flanking the xED motif (B3GNT2, POMGNT1, MGAT2, GGTA1, and B4GALT1). While no acceptor complex has been solved for Mfng, the overall fold of this protein is strikingly similar to B3GNT2 (rmsd of 2.4 Å for 191 Cα atoms) despite a sequence similarity of only 16%. In Mfng, the C-His (His256) that coordinates the divalent metal ion is positioned similar to B3GNT2, but is not conserved in sequence. Instead, the His residue originates from a different area of the Mfng structure (Fig. S4*A*) suggesting that other members of GT31 will likely conform to a similar protein fold compared with these two enzymes. GT31 members may also employ HV2 and HV3 residues and residues flanking the xED motif for their varied acceptor interactions.

### The B3GNT2 active site

**Figure 6:**
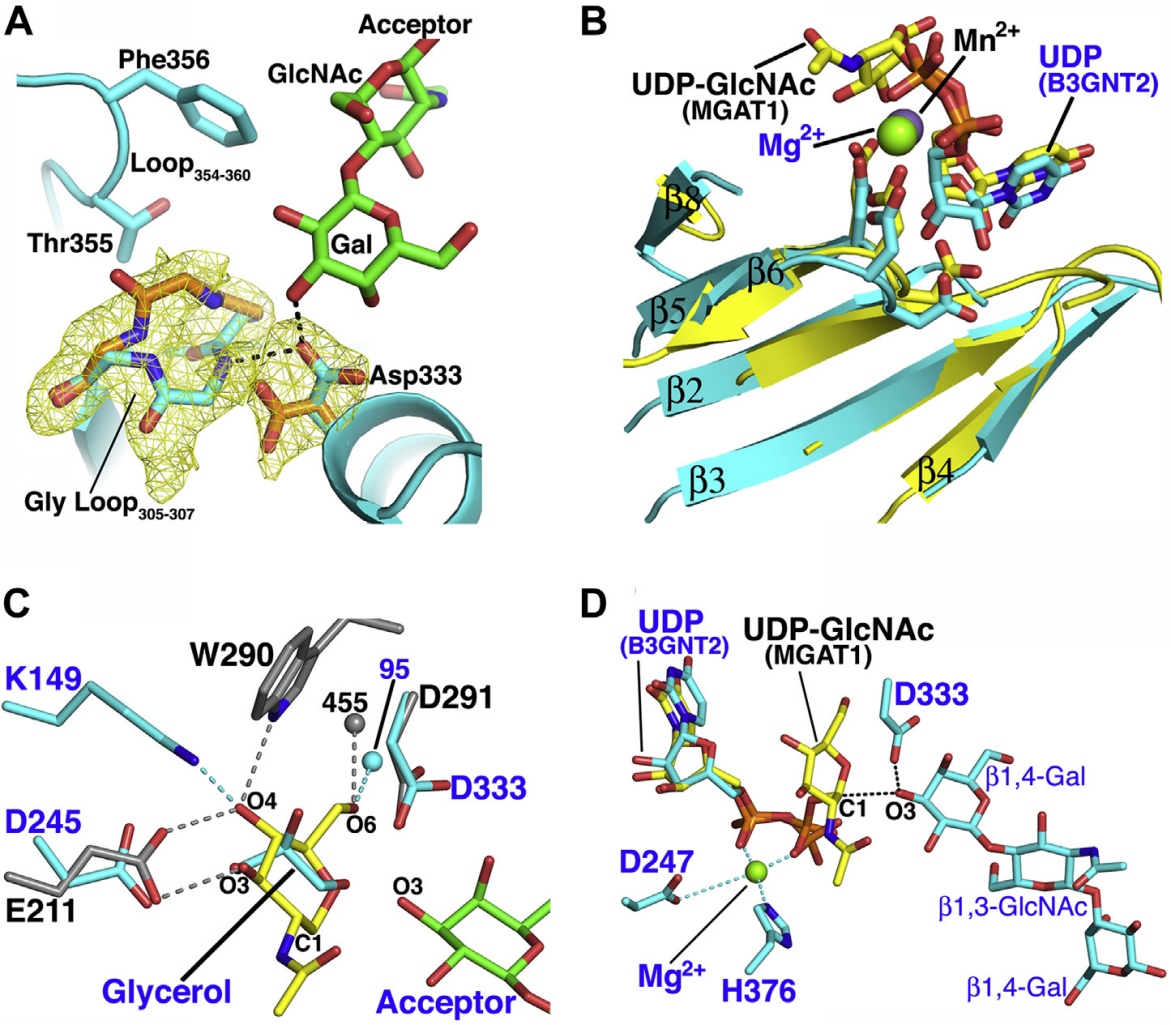
**Conformational changes in the B3GNT2 active site.***A*, Difference density map (F_o_–F_c_, *yellow mesh*) of the “Glycine-rich” loop (Gly-Loop_305:307_) and Asp333 in chain A of B3GNT2:UDP:Mg^2+^:LNnT (*cyan cartoon*), calculated at 1.85 Å and contoured at 3.0 σ. The map was calculated after omitting Gly-Loop_305:307_ and Asp333 from the refined coordinates of B3GNT2:UDP:Mg^2+^:LNnT and subjecting the model to simulated annealing. The active (*cyan*) and inactive (*orange*) conformations of Asp333 and Gly-Loop_305:307_ are shown. In the active conformation, the Asp333 carboxylate hydrogen bonds with the backbone amide of Gly303 and the nucleophile O3 of the acceptor Gal-β1,4- residue (*black dashed lines*). The nonreducing end Gal and GlcNAc of LNnT (*green*) and residue side chains from Loop_354-360_ that pack against the acceptor are also shown. *B*, the donor, UDP-GlcNAc (*yellow, stick*) modeled in the active site of B3GNT2:UDP:Mg^2+^:LNnT based on structural alignment with MGAT1:UDP-GlcNAc (1FOA). Superposition of the GT-A fold core β-sheets (β2, β3, β4, β5, β6, and β8) of MGAT1 (*yellow*, Mn^2+^ as *purple sphere*) and B3GNT2:UDP:Mg^2+^:LNnT (*cyan*, Mg^2+^ as *green sphere*) aligns the *DxD* motif (*sticks*), the metal ions, and the UDP moiety of the donor. *C*, the structural alignment of B3GNT2:UDP:Mg^2+^:LNnT (*cyan*) and MGAT1:UDP-GlcNAc (*gray*) to model the donor, UDP-GlcNAc, in the B3GNT2 active site. Hydrogen bonds between the MGAT1 side chains (*sticks*) and solvent water (*gray sphere*, numbered 455) and the GlcNAc (*yellow*, stick mode) are shown as *gray dashed lines*; the donor UDP has been omitted for clarity. In B3GNT2, a glycerol molecule occupies the same place as the modeled GlcNAc and the superposition places the O3 and O5 atoms of the GlcNAc in the same position as the O1 and O3 atoms of an ordered glycerol molecule. B3GNT2 residues (D245 and D333) and solvent water (*cyan sphere*, numbered 95) that are in position to conserve the hydrogen bonding interactions with the modeled GlcNAc are also shown. Interactions between O4 of the modeled GlcNAc and B3GNT2 residue K149 are depicted as *dashed lines* (*cyan*). *D*, The donor, UDP-GlcNAc (*yellow, sticks*), modeled in the active site of B3GNT2:UDP:Mg^2+^:LNnT (*cyan*). In B3GNT2, metal ion interactions involving the nucleotide diphosphate, side chains of H376 and D247 (last residue in the *DxD* motif) are depicted with *cyan dashes*. The proposed mechanism involves the catalytic base D333, deprotonating the O3 hydroxyl of the acceptor Gal-β1,4- residue. The deprotonation leads to the nucleophilic attack on the C1 atom of the UDP-GlcNAc donor (*black dashed lines*).

A comparison of the two active sites in the B3GNT2:UDP:Mg^2+^:LNnT dimer reveals a significant conformational change involving the conserved catalytic base (Asp333) in the “xED” motif. In chain A, the electron density for Asp333 shows that the rotamer can be modeled with χ_1_ torsion angles of –64° and 57° (60% and 40% occupancies, respectively), while chain B only contains the –64° conformation (Fig. 6*A*). The –64° χ_1_ torsion angle positions the carboxylate to act as a base for deprotonating the nucleophilic O3 of the Gal-β1,4- residue of the acceptor (Fig. 6*A*). In this conformation, the Asp333 carboxylate also accepts a hydrogen backbone amide of Gly306, which is part of the “Glycine-rich” loop (Gly-Loop_305:307_). The χ_1_ torsion angle of 57° directs the carboxylate away from the active site and represents an inactive conformation. To make space for this rotameric change, the Gly-Loop_305:307_ shifts by 4.3 Å and extends into the active site (Fig. 6*A*). We believe that this inactive conformation is an artifact of using UDP as a donor analog; it is likely that the GlcNAc of the authentic donor would sterically prevent the repacking of the Gly-Loop_305:307_, which in turn would lock Asp333 in the active state. In the acceptor-free B3GNT2:UDP:Mg^2+^ and SeMet-B3GNT2:UDP:Mg^2+^ complexes, the Asp333 and Gly-Loop_305:307_ are only observed in the inactive conformation despite the fact that the latter structure was solved in a crystal form that is isomorphous to the B3GNT2:UDP:Mg^2+^:LNnT complex. This suggests acceptor binding contributes to the stabilization of the active state conformation of the enzyme.

### Modeling UDP-GlcNAc in the B3GNT2 active site

The structure of an intact UDP-GlcNAc donor complex for B3GNT2 could not be obtained because of hydrolysis of the sugar donor. However, an intact donor complex is available for the homologous inverting β1,2-GlcNAc transferase, MGAT1 (MGAT1:UDP-GlcNAc:Mn^2+^, PDB 1FOA) (39, 43). Despite a low sequence identity of only 7%, B3GNT2 and MGAT1 superimpose 169 equivalent Cα atoms with an rmsd of 3.5 Å. The superposition shows that the core GT-A fold, many active site residues, *DxD* motif, metal ion, and nucleotide donor binding site are structurally conserved but do not align ideally. To model the intact UDP-GlcNAc donor in the B3GNT2:UDP:Mg^2+^:LNnT active site, a subsequent superposition of the GT-A fold core β-sheets (β2, β3, β4, β5, β7 and β8) of MGAT1 (1FOA) and B3GNT2:UDP:Mg^2+^:LNnT was carried out to refine the alignment between the two structures (rmsd of 1.2 Å for 36 corresponding C*α* atoms) (Fig. 6*B*). The superposition aligns the *DxD* motif, the metal ion, and the UDP moiety of the donor (Fig. 5*B*). The superposition also places the O3 and O5 atoms of the GlcNAc in the same position as the O1 and O3 atoms of an ordered glycerol molecule that was identified in the active site of the B3GNT2 crystal structure (Fig. 6*C*). This observation supports our simple modeling experiment, since it is not unusual to observe ordered glycerol, waters, or related molecules occupying the same position as the sugar hydroxyls of substrate molecules in enzyme active sites (44, 45, 46). The superposition also shows that the architecture of the B3GNT2 active site complements both the shape and hydrogen bonding requirements of the modeled GlcNAc with no significant steric clashes (Fig. 6*D*). The only conserved hydrogen bonds are formed by the GlcNAc O3 and O4 hydroxyls, which donate to the carboxylate of the first residue in the *DxD* motif (Glu211 and Asp245 in MGAT1 and B3GNT2, respectively). In MGAT1, the GlcNAc O4 atom also accepts a hydrogen from the indole nitrogen of Trp290, which is replaced by Asp332 in B3GNT2 (Fig. 6*C*). The Asp332 is unlikely to donate a hydrogen to the O4 atom, but our modeling suggests that this interaction can be satisfied by the amine group of Lys149 in B3GNT2, which is not conserved in MGAT1 (Fig. 6*C*). The remaining hydrogen bond is formed between the GlcNAc O6 hydroxyl and an ordered water molecule that is similarly positioned in both the B3GNT2 and MGAT1 active sites (W95 and W455, respectively). Additional support for the placement of the GlcNAc comes from observation that the C1 atom is ideally positioned for a nucleophilic attack by the O3 hydroxyl of the acceptor Gal-β1,4-, which is consistent with the proposed inverting catalytic mechanism (Fig. 6*D*). The superposition of B3GNT2 and MGAT1 also places the corresponding catalytic bases (Asp333 and Asp291, respectively) in the same position to deprotonate the acceptor nucleophile. It is notable that the inactive conformation of the Gly-Loop_305:307_ would introduce a steric clash with the modeled GlcNAc (not shown). Finally, this model is supported by site-directed mutagenesis of the predicted interacting residues in B3GNT2, which results in a significant loss in enzyme activity (14,100-, 14,300-, 3680-, and 15,800-fold reduction in k_cat_/K_m_ for D245A, K149A, D332A, and D333A mutants, respectively, Table S3).

### Comparison of the B3GNT2 catalytic mechanism with other GT-A inverting enzymes

Our modeled conformation of the sugar donor is also consistent with what is observed across the GT-A fold family. Since most glycosyltransferases are prone to hydrolysis of the sugar donor, only a limited number of structures are available that contain the intact donor substrate: among these are the inverting enzymes B4GALT1 (GT7) (47), MGAT1 (GT13) (39), XylT1 (GT14) (48), B3GAT3 (GT43) (49) and the retaining enzymes GGTA1 (GT6) (50), XxylT1 (GT8) (51), GALNT2 (GT27) (52), and Mgs (GT78) (53). In all of these examples, the sugar packs against residues in and near the N-terminus of the α10 helix (Fig. 4*C*). Superimposing the core elements of the GT-A fold shows that the position and conformation of the nucleotide sugar deviate significantly between the enzymes, presumably due to the sequence variation in the binding site and throughout the protein. However, superimposing the ribosyl diphosphate of the nucleotide and the main chain atoms of the residue corresponding to the catalytic base in B3GNT2 shows that the C1 atoms of the donor sugars cluster in almost the same position regardless of the identity of the donor sugar (Fig. S5*A*). More importantly, the structural overlay shows that the relative positions of the acceptor nucleophile and the catalytic base correlate with the specific enzyme mechanism (Fig. S5*B*) as has been noted in a previous study (38). For example, in the metal-dependent inverting glycosyltransferases that employ an Asp side chain as catalytic base, the nucleophilic hydroxyls of the acceptor glycans form a tight cluster with a radius of ∼1 Å (Fig. S5*B* and Table S2). The highly conserved positioning is surprising given the variation in the structural and chemical context of the acceptor nucleophile (Fig. S5*C*). For example, in B3GNT2, the nucleophile is the O3 of a Gal residue (10), in Mfng it is the O3 of a Fuc residue (30), enzymes MGAT1, MGAT2, and POMGNT1 use the O2 of a Man (54, 55, 56, 57), and B4GALT1 attacks with the O4 of a Gal residue (58).

## Discussion

The CAZy GT31 family of enzymes is the largest glycosyltransferase family in mammals (25 of >220 human glycosyltransferase genes (24)) and contains a diverse collection of donor and acceptor specificities (32). This family is also among the least studied at the structural level, with the Mfng:UDP:Mn^2+^ complex as the sole representative structure in this family (30). The GT31 family also includes the enzymes responsible for poly-LacNAc biosynthesis, but the characterization of these enzymes responsible for poly-LacNAc biosynthesis has been surprisingly complicated. In fact, the first putative poly-LacNAc synthase, B3GNT1 (CAZy GT49), was incorrectly identified (59) and has since been shown to be a β1,4-galacturonosyl transferase involved in matriglycan synthesis and renamed B4GAT1 (CAZy GT49) (60, 61). The correct identification of the true poly-LacNAc synthases resulted from the cloning of eight additional genes (B3GNT2-B3GNT9) in CAZy GT31 (4, 10, 12, 18, 35, 62, 63, 64), each displaying distinctive capabilities for β1,3GlcNAc addition to different glycan classes (25, 26). Because B3GNT2 is the most abundant poly-LacNAc synthase and has the largest tissue distribution, it is believed to be the primary poly-LacNAc synthase in mammalian organisms. Our goal was to understand how B3GNT2 recognizes its sugar donor and glycan acceptor substrates and catalyzes poly-LacNAc extension.

Similar to Mfng, B3GNT2 is a metal-dependent GT-A fold inverting enzyme that binds its divalent cation through a conserved *DxD* motif. Previous studies indicated that B3GNT2 had similar levels of activity toward poly-LacNAc substrates with varying numbers of LacNAc units (10), suggesting that the enzyme only recognizes terminal Galβ(1–4)-GlcNAc- structures. The crystal structure of the B3GNT2:UDP-Mg^2+^:LNnT complex confirmed this hypothesis by demonstrating that the enzyme interacts exclusively with the nonreducing terminal LacNAc unit (Fig. 5). The structure also shows that the B3GNT2 acceptor binding site will not accommodate modifications to the terminal Galβ(1–4)-GlcNAc- of the acceptor, which explains the lack of recognition of Type 1 acceptors (Galβ(1–3)-GlcNAc-), fucosylation of either the Gal or GlcNAc residues (H Type 2 or Lewis X antigen structures), or sialylation of the nonreducing Gal residue (sialyl Lewis X antigen) (19, 35, 62, 65).

Sedimentation velocity, SEC-MALS, and structural analysis (Figs. 2, *C*–*D* and 3*A*) all indicate that B3GNT2 exists as a dimer *in vivo* where it is tethered to the luminal face of the Golgi complex by an N-terminal transmembrane domain (Fig. 3*A*). The twofold symmetry axis for the dimer places both active sites on the same face of the enzyme, which raises the possibility of increased avidity for poly-LacNAc extensions through interactions with multiple termini on the same glycan structure. However, the two active sites are separated by ∼35 Å, which means that only highly extended multiantennary N-glycan structures (>5 poly-LacNAc repeats on a tri-antennary N-glycan) could bridge between the two homodimeric active sites, suggesting multivalent modification of a single glycan is unlikely (Fig. S6). Still, it is possible that multivalent enzymatic modifications of closely spaced N- or O-glycan structures on a the same glycoprotein substrate can occur.

**Figure 7:**
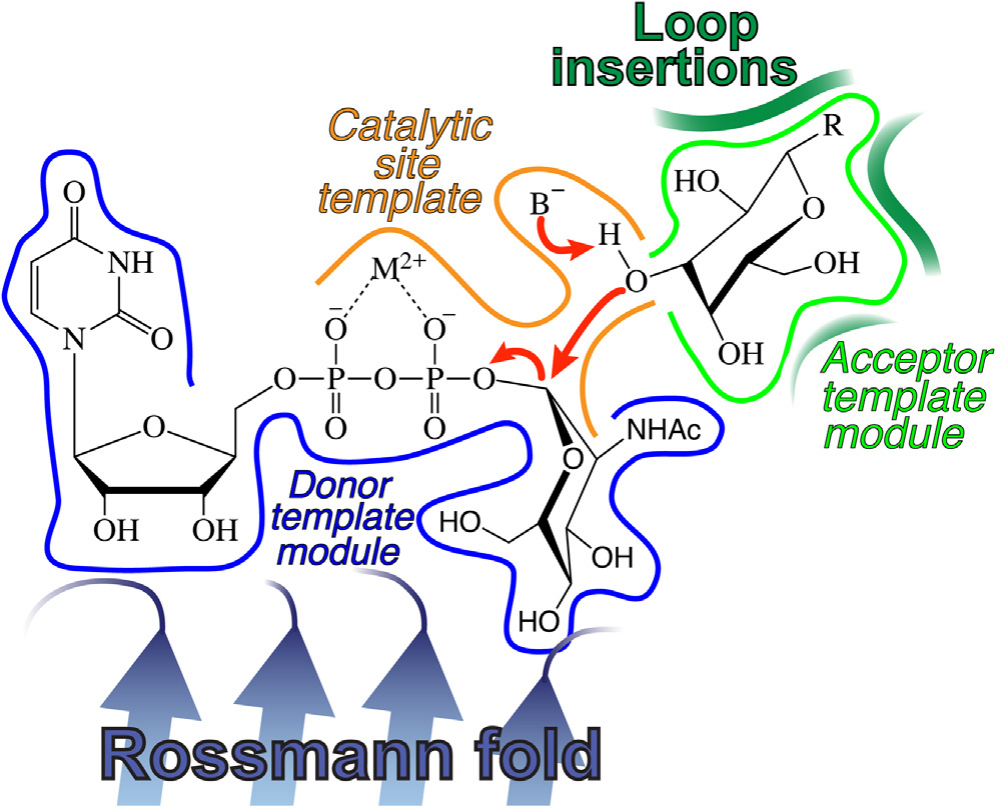
**The modular assembly of the active site of metal-dependent, inverting GT-A fold glycosyltransferases.** A generalized model for the active site of a GT-A fold metal-dependent, inverting glycosyltransferase is depicted based on the structure of the proposed B3GNT2 catalytic mechanism (*red arrows*). Residues in the “Donor template module” (*blue outline*) facilitate sugar–nucleotide interactions and define the specificity for nucleotide and donor sugar. The “Acceptor template module” (*green outline*) recruits the extended glycan acceptor and appropriately positions the hydroxyl nucleophile using loop insertions into the core GT-A fold. The “Catalytic site template” (*orange outline*) positions the catalytic base relative to the C1 of the donor sugar and hydroxyl nucleophile to specify the inverting or retaining mechanism for group transfer.

The B3GNT2:UDP-Mg^2+^:LNnT complex also provides insights into glycosyltransferase substrate recognition and evolution. B3GNT2 employs a classical *DxD* motif for coordinating the enzyme-bound divalent cation that anchors sugar donor interactions. Similar to other GT-A fold glycosyltransferases, the nucleotide sugar binding site in B3GNT2 is built from a collection of conserved structural elements, including Loop_β2-α4_, Loop_β4-α6_, and Loop_β5-β6_ (Figs. 4*C*, 5 and 7). Despite the conserved architecture of the donor subsite among the GT-A fold enzymes, there is surprisingly little sequence conservation of the residues that directly interact with the sugar donor. Broadly conserved features are limited to the *DxD* motif in the metal-dependent enzymes, the catalytic base in the xED motif for enzymes that use an inverting catalytic mechanism, a “Glycine-rich” loop facing the donor binding site dominated by short side chains, and the use of a C-terminal His residue for metal coordination (37). For example, both B3GNT2 and MGAT1 bind UDP-GlcNAc donors in similar conformations and conserve the *DxD* motif, the “Glycine-rich” loop and the catalytic base, but the C-His residue for metal coordination is lacking in MGAT1. The remaining interactions between the two enzymes and their UDP-GlcNAc donor sugars are similar, but employ different amino acids (Fig. 6*C*). Other GT-A fold enzymes display a similar degree of sequence and structural plasticity in binding the same donor sugar, which complicates efforts to predict substrate specificity (Fig. 4*C*).

By comparison, the acceptor binding subsite in B3GNT2 is minimally conserved among GT-A fold enzymes. The structural elements for acceptor interactions are the most complex modular features in the GT-A fold enzymes and have been the hardest to predict without structural information from enzyme:acceptor complexes. Prior sequence-based (37) and structural alignment (38, 42) studies on GT-A fold enzymes identified three positions of hypervariable loop insertion (HV1, HV2, and HV3) into the conserved GT-A fold core that can be used for assembly of acceptor binding sites. A significant expansion of any of these three loops is a potential indicator of their contributions to acceptor interactions. For example, topology diagrams for a small collection of GT-A fold enzymes (Fig. S3) illustrate the varied positions of acceptor binding residues within these loops. Our present work on B3GNT2 shows that HV2 (Loop_β7-β8_) and HV3 (Loop_β7-C-term_) (Fig. 5 and Fig. S3) form an acceptor binding cleft that tightly encloses the relatively small terminal LacNAc unit of the acceptor glycan. In contrast, the hypervariable loops in other GT-A fold enzymes can form extended acceptor binding subsites to accommodate significantly larger branched glycan acceptors (45) (Fig. S3). The structural elements encompassing the xED motif and flanking residues also interact with the acceptor, which acts as an additional structural motif for the evolution of new specificities. Thus, glycosyltransferases undergo rapid evolution through loop insertions and sequence variation at restricted positions to evolve new specificities for the synthesis of diverse glycan structures.

Finally, the third component of the B3GNT2 structure is the catalytic site. Similar to other GT-A fold enzymes (38), the assembly of the donor and acceptor binding subsites in B3GNT2 results in the appropriate positioning of the Gal O3 hydroxyl nucleophile adjacent to the Asp333 catalytic base and C1 of the GlcNAc donor to facilitate direct in-line S_N_2 nucleophilic attack, with the displacement of the nucleotide diphosphate as the leaving group. It is striking that the relative positions of the donor sugar C1, the acceptor hydroxyl oxygen, and the catalytic base are so well conserved among inverting enzymes given the weak conservation of donor sugar interactions and the tremendous diversity of acceptor structures and binding subsite loops. The evolutionary pressures to optimally align the donor, acceptor, and catalytic base are satisfied by small shifts in the positioning of the donor sugar and the acceptor glycans. This apparent plasticity in substrate binding likely explains the low level of sequence conservation observed in the substrate binding sites.

Metal-dependent retaining GT-A fold enzymes also conserve the use of a *DxD* motif, positioning of the sugar nucleotide donor relative to the Rossmann fold core, and the same use of hypervariable loop insertions for assembly of acceptor binding sites (38, 50). However, they evolved an alternative positioning for the acceptor hydroxyl nucleophile away from the catalytic base position used by inverting enzymes to a position adjacent to the donor β-phosphate oxygen. Deprotonation of the acceptor hydroxyl group by the phosphate oxygen commonly leads to a dissociative S_N_i-type retaining mechanism for sugar transfer (38). Thus, it appears that the repositioning of the acceptor hydroxyl is the major difference in the switch between inverting and retaining GT-A fold enzymes (38).

A surprising observation from a recent analysis of GT-A fold enzyme sequences was that these genes evolved to interconvert in catalytic mechanism (inverting vs. retaining) at multiple independent points during their duplication and diversification (37). These catalytic interconversions can only be achieved through evolutionary drift in the position of the acceptor hydroxyl nucleophile relative to the sugar donor followed by selection for the resultant catalytic activities. Consistent with this hypothesis, we observed small but significant variations in position for the sugar donor, nucleophile hydroxyl, and catalytic base relative to the Rossmann fold core for the diverse collection of GT-A fold enzymes (Fig. S5). While coordinated shifts in position of the donor, acceptor, and catalytic base are tightly maintained among the inverting enzymes, the relative positions of donor and acceptor appear to be decoupled for retaining enzymes, perhaps to accommodate the proposed substrate-assisted deprotonation that leads to the sugar transfer with retention of anomeric configuration.

Our analysis of the B3GNT2 structure reveals a recurring theme in the evolution of GT-A fold glycosyltransferases; substrate specificity is achieved through a combination of divergent and convergent evolution of distinct template modules for donor and acceptor interactions and catalysis built upon a common structural architecture (Fig. 7). Similar modular active site structures are anticipated for the other glycosyltransferase classes (GT-B and GT-C fold enzymes), and it is these modular structural elements that provide the three-dimensional enzymatic templates that produce the glycan diversity observed in biological systems.

## Experimental procedures

### B3GNT2 expression and selenomethionine labeling

A protein expression construct encoding the catalytic domain of human β-1,3-N-acetylglucosaminyltransferase 2 (UniProt Q9NY97, residues 34–397) was generated by PCR from a Mammalian Gene Collection clone followed by Gateway recombination into the pDONR221 vector (32). The PCR amplification extended the truncated B3GNT2 coding region by inclusion of flanking Gateway att1 recombination sites as well as an extension of the NH_2_-terminus of the coding region with a TEV protease recognition site as previously described (32). Gateway LR recombination of the TEV-B3GNT2-pDONR221 vector with the mammalian Gateway-adapted expression vector (pGEn2-DEST) generated the B3GNT2-pGEn2 expression construct. The fusion protein construct encodes an NH_2_-terminal signal sequence, 8xHis tag, AviTag, “superfolder” GFP, the TEV protease recognition site, and the truncated B3GNT2 coding region behind a CMV promoter (32). This B3GNT2-pGEn2 expression vector was used for transient transfection of either FreeStyle 293-F cells (ThermoFisher Scientific) or HEK293S (GnTI-) cells (ATCC) in suspension culture using polyethylenimine (linear 25 kDa PEI, Polysciences, Inc) as transfection reagent (31, 32). The cultures were diluted 1:1 with culture medium containing 4.4 mM valproic acid (2.2 mM final concentration) 24 h after transfection, and protein production was continued for a further 5 days at 37 °C. For metabolic labeling of HEK293S (GnTI-) cells with selenomethionine (SeMet), cells were transfected as described above and 12 h after transfection, the medium was exchanged for custom methionine-free Freestyle 293 expression medium (Thermo Fisher) for 6 h to deplete the methionine pools. The cultures were subsequently resuspended in methionine-free Freestyle 293 expression medium containing 60 mg/liter SeMet, and protein production was continued for further 4–5 days at 37 °C (31, 32).

### B3GNT2 purification

The conditioned culture medium was loaded onto a Ni^2+^-NTA Superflow (Qiagen) column pre-equilibrated with 20 mM HEPES (pH 7.4), 300 mM NaCl and 20 mM imidazole (Buffer A). The column was washed with three column volumes of Buffer A followed by three column volumes of Buffer A containing 40 mM imidazole, and eluted with Buffer A containing 300 mM imidazole, pH 7.0. The eluted fusion protein was concentrated to approximately 2 mg/ml. Both purified His/GFP-tagged TEV protease and EndoF1, expressed in *E. coli* (31, 32), were added to the concentrated protein sample in the Ni-NTA elution buffer at a ratio of 1:10 relative to the fusion protein and incubated at 4 °C for 24 h to cleave the fusion tag and glycans. The cleaved B3GNT2 was further isolated from the fusion tag, His-tagged TEV protease, and EndoF1 by Ni^2+^-NTA chromatography. The protein was then concentrated and further purified on a Superdex 75 column (GE Healthcare) with a buffer containing 20 mM HEPES (pH 7.4), 200 mM NaCl, 60 mM imidazole. Peak fractions of B3GNT2 were collected, and the buffer was exchanged into buffer containing 10 mM HEPES (pH 7.0), 50 mM NaCl, 200 mM betaine, and 10% glycerol and concentrated by ultrafiltration to 30 mg/ml for crystallization.

### Ligands and chemicals

UDP and UDP-GlcNAc were obtained from Sigma-Aldrich (St Louis, MO). Lacto-N-neotetraose (Gal(β1-4)GlcNAc(β1-3)Gal(β1-4)Glc, LNnT) was obtained from Carbosynth (San Diego, CA).

### B3GNT2 mutagenesis and enzyme assays

In order to confirm the interactions between the sugar donor, acceptor, and enzyme, mutagenesis studies were performed on several active site residues. Site-directed mutagenesis using the Q5 Site-Directed Mutagenesis Kit (New England Biolabs, Ipswich, MA) was performed based on the structure of B3GNT2. Mutant enzymes were generated by transient transfection of HEK293-F cells, and enzyme activity was determined following purification by Ni-NTA Superflow chromatography. Formation of UDP as a by-product of the glycosyltransferase reaction was measured using the UDP-Glo glycosyltransferase Assay (Promega, Madison, WI) according to manufacturer’s instructions. The reactions were performed in a 5 μl volume containing 100 mM HEPES (pH 7.4), 2 mM MnCl_2_, 0.1 mM UDP-GlcNAc (donor), 0.5 mM LNnT (acceptor), 1 mg/ml BSA, and purified wild-type or mutant forms of GFP-B3GNT2 (0.4–2000 ng depending on enzyme activity of the mutant). After incubation for 1 h at 37 °C, the reactions were stopped by mixing with 5 μl of UDP detection reagent. The samples were transferred into an opaque 384-well plate (Corning, Corning, NY) and incubated for 60 min. After incubation, luminescence measurements were performed using a GloMax Multi Detection System plate reader (Promega). Luminescence values were compared with a standard curve for quantification of UDP, and steady-state parameters of k_cat_, K_m_, and V_max_ were calculated by fitting initial velocities using nonlinear curve fitting in GraphPad Prism 6 (GraphPad Software).

### Size-exclusion chromatography coupled with multiangle light scattering (SEC-MALS)

Purified B3GNT2 (20 μl at 1 mg/ml) was analyzed by SEC-MALS on a Superdex 75 gel filtration column (GE Life Sciences) in a buffer containing 25 mM HEPES (pH 7.4), 150 mM NaCl, 0.02% NaN_3_. In-line light scattering was measured using a MiniDAWN TREOS detector (Wyatt Technology) and differential refractive index using an Optilab rEX detector (Wyatt Technology). Data were analyzed using Astra 6 software (Wyatt Technology).

### Analytical ultracentrifugation

Sedimentation velocity studies were conducted using a Beckman Coulter Optima XLA analytical ultracentrifuge at a temperature of 20 °C. B3GNT2 (12 μM) was exchanged into a buffer containing 250 mM NaCl and 20 mM HEPES (pH 7.4). The sample and reference were loaded into 12 mm double-sector Epon centerpieces with quartz windows, placed in a four-hole An-60 Ti rotor, and run at a speed of 50,000 rpm. Data were collected at a wavelength of 280 nM using a radial step size of 0.003 cm. The partial specific volume of 0.73985 ml/g was calculated from the amino acid sequence of the B3GNT2 catalytic domain. SEDNTERP was used to calculate the density (1.0101 g/ml) and the viscosity (0.0104 P) for the buffer (66). SEDFIT was used to analyze and model the raw sedimentation data (67). Modeled data were fit as continuous sedimentation coefficient c(s) distributions using the baseline, meniscus, frictional coefficient, systematic time-invariant noise, and radial-invariant noise. The c(s) analyses were restrained by maximum entropy regularization at *p*-value=0.95 confidence interval. The weight-averaged sedimentation coefficient for each species was determined by integrating each peak in the c(s) distribution and reported as the S-value. The theoretical S-value for the B3GNT2 dimer was calculated from the crystal structures using HydroPro (68).

### Crystallization and data collection

The catalytic domain of selenomethionine derivatized B3GNT2 (residues 34–397, 8 mg/ml) in 5 mM UDP, 5 mM MgCl_2_, and storage buffer (10 mM HEPES pH 7.0, 50 mM NaCl, 200 mM betaine, and 10% glycerol) was screened for crystallization conditions using a TTP Labtech Mosquito Crystal robot and optimized using hanging drop vapor diffusion with 2 μl drops (1:1 protein:reservoir ratio). Crystals grew in less than a week from a reservoir of 16% PEG3350, 16% ethylene glycol, and 100 mM HEPES (pH 7.5). Crystals were transferred to the reservoir solution supplemented with 5 mM UDP, 5 mM MgCl_2_, and 6% cryoprotectant (2:1 DMSO and glycerol). SeMet-B3GNT2:UDP-Mg^2+^ crystallized in P2_1_2_1_2_1_ and diffracted to 1.55 Å resolution (Table 1).

For the B3GNT2:UDP-Mg^2+^ complex, 20 mg/ml of protein in storage buffer containing 2 mM UDP and 5 mM MgCl_2_ grew crystals overnight from a reservoir solution of 11% PEG3350, 12% ethylene glycol, and 100 mM HEPES (pH 7.5). Crystals were transferred to the reservoir solution supplemented with 2 mM UDP-GlcNAc, 5 mM MgCl_2_, and 12% cryoprotectant (2:1 DMSO and glycerol). The B3GNT2:UDP-Mg^2+^ complex crystallized in P2_1_ and diffracted to 2.04 Å resolution (Table 1).

The B3GNT2 acceptor complex was crystallized using 20 mg/ml of protein in storage buffer containing 5 mM UDP, 5 mM MgCl_2_, and 10 mM LNnT. Crystals grew overnight from a reservoir solution of 24% PEG6000, 6% ethylene glycol, and 100 mM Tris (pH 8.5). Crystals were cryoprotected using the reservoir solution supplemented with 5 mM UDP, 5 mM MgCl_2_, 10 mM LNnT, and 13% cryoprotectant (2:1 DMSO and glycerol). The B3GNT2:UDP-Mg^2+^:LNnT complex crystallized in P2_1_2_1_2_1_ and diffracted to 1.85 Å resolution (Table 1).

All cryo-protected crystals were flash cooled in liquid nitrogen, and X-ray data were collected at the SER-CAT 22-ID beamline at the Argonne National Laboratory using a Rayonix 300HS detector and processed using XDS (69). Five percent of the data was set aside for cross validation.

### Phasing and refinement

Data from the selenomethionine-derivatized protein (SeMetB3GNT2) was used for phasing with single-wavelength anomalous diffraction. B3GNT2 has six methionine residues, and a data set with 7.6-fold redundancy using a 0.95 Å wavelength was collected to maximize the anomalous signal. Ten selenium sites were located using the Hybrid Substructure Search in Phenix (70). The calculated protein phases had a figure of merit of 0.34, and iterative cycles of automated model building and density modification using AutoSol (71) produced the initial model for SeMet-B3GNT2:UDP-Mg^2+^. The NH_2_-terminus (residues 34–56, 73–89 in chain A and 34–51, 72–90 in chain B, respectively) and the COOH-terminus (residues 395–397 in chain A) were disordered and were left unmodeled. Due to the COOH-terminal disorder in chain A, the disulfide bond involving Cys397 was also left unmodeled. Further rounds of automated refinement in Phenix (70) and iterative manual fitting using Coot (72) produced the final model (Table 1). The B-factors were refined using TLS (73).

The 1.85 Å resolution crystal structure of B3GNT2:UDP-Mg^2+^:LNnT was solved using molecular replacement in space group P2_1_2_1_2_1_ with B3GNT2:UDP-Mg^2+^ as the search model (71) (Table 1). The NH_2_-terminus (residues 34–53, 72–90 in chain A and 34–55, 72–90 in chain B, respectively) and COOH-terminal residues 393–397 in chain B were disordered and left unmodeled. The B3GNT2:UDP-Mg^2+^:LNnT model was refined like SeMet-B3GNT2:UDP- Mg^2+^, and the statistics are reported in Table 1.

During refinement, geometrical restraints were used to constrain monosaccharides with weak electron density in the lowest energy chair conformation (^4^C_1_) (70, 74). The linkage torsion angles for the glycosidic bonds (ϕ, ψ) in SeMet-B3GNT2:UDP-Mg^2+^ and B3GNT2:UDP-Mg^2+^:LNnT complexes were mapped using the Carbohydrate Ramachandran Plot (CaRP) and fall into the energetically preferred areas of the GlyTorsion plot (75).

### Structural analysis

The structure-based sequence alignment of recombinant human B3GNT2 with the catalytic domain of the *Mus musculus* O-fucosylpeptide β-1,3-N-acetylglucosaminyltransferase from GT31 (Manic fringe, Mfng, PDB entry 2J0B) and the murine leukocyte-type core 2 β-1,6-N-acetylglucosaminyltransferase from GT14 (Gcnt1, PDB code 3OTK) was carried out using the Dali Pairwise comparison tool (76).

## Data availability

The atomic coordinates and structure factors have been deposited in the Protein Data Bank, wwpdb.org (PDB ID codes 6WMM, 6WMN, and 6WMO). All other data are included in this article.

## Conflict of interests

The authors declare that they have no conflicts of interest with the contents of this article.
