# Supplementary material for: Comparison of human poly-N-acetyl-lactosamine synthase structure with GT-A fold glycosyltransferases supports a modular assembly of catalytic subsites
Source: J Biol Chem. 2020 Dec 3;296:100110. doi: 10.1074/jbc.RA120.015305 (PMC7948508; doi:10.1074/jbc.RA120.015305)
Supplement: Figures and Tables [file mmc1.pdf]

Supporting Information

Comparison of human poly-*N*-acetyl-lactosamine synthase structure with GT-A fold glycosyltransferases supports a modular assembly of catalytic subsites

Renuka Kadirvelraj[1,†] Jeong-Yeh Yang[2,†], Hyun Woo Kim[2], Justin H. Sanders[2], Kelley W. Moremen[1,2,*] and Zachary A. Wood[2,*]

[1]Department of Biochemistry & Molecular Biology, University of Georgia, Athens, GA 30602, USA, and [2]Complex Carbohydrate Research Center, University of Georgia, Athens, GA 30602-4712, USA.

[†] These authors contributed equally to this work.

[*]Correspondence: Kelley Moremen, email: moremen@uga.edu and Zachary A. Wood, email: zaw@uga.edu

This Supporting Information includes:

Supplementary Table S1: B3GNT2 dimer interface parameters calculated using PISA

Supplementary Table S2: Proteins with structurally similar GT-A fold to B3GNT2 based on a DALI search of the PDB database.

Supplementary Table S2: Kinetic parameters for wild type human B3GNT2 and mutants using UDP-GlcNAc as donor and LNnT as acceptor substrate.

Supplementary Figure S1: Sequence and structural alignment of B3GNT2 with related GTs.

Supplementary Figure S2: The *N*-glycosylation at residue Asn219 in SeMet-B3GNT2:UDP-Mg^{2+}.

Supplementary Figure S3: Comparison of acceptor template modules in inverting and retaining GT-A fold enzymes.

Supplementary Figure S4: Interactions of the UDP donor-analog in B3GNT2 and other GT-A fold GTs.

Supplementary Figure S5: Relationship of the sugar donor and acceptor nucleophile hydroxyl positions in inverting and retaining GT-A fold enzymes.

Supplementary Figure S6: The two-fold axis of symmetry for the B3GNT2 dimer indicating that the active sites are on the same face of the homodimeric enzyme.

Supplementary Table S1: B3GNT2 dimer interface parameters calculated using PISA

Interface residues	Interface area	Δ^iG	Δ^iG	Hydrogen bonds & salt bridges	Polar residues in interface	Non-polar residues in interface
	Å^2	*kcal/mole*	*P-value*			
24	924	-17.5	0.007	6	Q159, E163, D189, N190, H191, D193, D196, E201, K204, H205, Q381, D385, S388	P153, F155, A156, A160, P187, P192, L194, M197, F200, P380, I384

Supplementary Table S2: Proteins with structurally similar GT-A fold to B3GNT2 based on a DALI search of the PDB database[a]

GT-A fold glycosyl transferases with representative donor and acceptor bound crystal structures									
**Enzyme (Source)**	***CAZy family***	**Enzyme donor**	**Enzyme acceptor**	**Donor/donor analog and acceptor in crystal structures**	**PDB codes[b]**	**Mechanism (Metal dependence)**	**Sequence identity[a] (%)**	**RMSD (Å)**	**Aligned residues[c]**
BcsA (*R. sphaeroides*)	GT2	UDP-Glc	Glc unit of Cellulose	UDP, Cellulose 17-mer polymer	4P00	Inverting (Yes)	9	5.7	185
GGTA1 (*B. taurus*)	GT6	UDP-Gal	Gal of Galβ1,4-GlcNAc-R	UDP-Gal, Lactose	5NRB	Retaining (Yes)	8	4.2	184
BoGT6A (*B. ovatus*)	GT6	UDP-GalNAc	Gal of 2'-Fucosyl Lactose/LacNAc	UDP-GalNAc, 2'-Fucosyllactose	4CJC, 4AYJ	Retaining (No)	10	4.1	191
B4GalT1 (*B. taurus*)	GT7	UDP-Gal	Terminal GlcNAc of polysaccharides	UDP-Gal, Chitobiose	1TW1, 1TW5	Inverting (Yes)	9	3.6	149
XxylT1 (*M. musculus*)	GT8	UDP-Xyl	*O*-linked Glc in protein EGF-like repeats	UDP-Glc, Xyl-β1,3-Glc-EGF	4WMA	Retaining (Yes)	8	4.6	185
POMGnT1 (*H. sapiens*)	GT13	UDP-GlcNAc	*O*-linked terminal Man on glycoproteins	UDP, Mannosyl peptide	5GGI	Inverting (Yes)	8	4.2	173
Gcnt1 (*M. musculus*)	GT14	UDP-GlcNAc	Core 1 O-glycan	UDP, Gal-β1,3-GalNAc	3OTK, 2GAM	Inverting (No)	4	3.1	214
XylT1 (*H. sapiens*)	GT14	UDP-Xyl	*O*-linked Glc in protein EGF-like repeats	UDP-Xyl, Peptide QEEEGSGGGQGG	6EJ7, 6EJ8	Inverting (No)	6	3.7	227
Kre2 (*S. cerevisiae*)	GT15	GDP-Man	Lipid-linked core oligosaccharide	GDP, Methyl-α-Man	1S4P	Retaining (Yes)	6	3.4	176
Mgat2 (*H. sapiens*)	GT16	UDP-GlcNAc	α-Man of di-antennary complex *N*-glycans	UDP, GlcNAc-β1,2-Manα1,3-Man	5VCM, 5VCS	Inverting (Yes)	12	3.5	190
GalNT2 (*H. sapiens*)	GT27	UDP-GalNAc	*O*-linked Ser/Thr of proteins	UDP-GalNAc, Glycopeptide	4D0T, 5AJP	Retaining (Yes)	12	3.2	169
XT1 (*A. thaliana*)	GT34	UDP-Xyl	Glc of Xyloglucan	UDP, Cellohexaose	6BSW	Retaining (Yes)	10	3.6	192
B3GAT1 (*H. sapiens*)	GT43	UDP-GlcA	Gal-β1,4-GlcNAc- of HNK-1 epitope	UDP, Gal-β1,4-GlcNAc	1V84	Inverting (Yes)	14	3.2	167
B3GAT3 (*H. sapiens*)	GT43	UDP-GlcA	Gal-β1,3-Gal-β1,4-Xyl linked to *O*-glycans	UDP-GlcA, Gal-β1,3-Gal-β1,4-Xyl	1KWS, 1FGG	Inverting (Yes)	13	3.2	165
EXTL2 (*M. musculus*)	GT64	UDP-GlcNAc	*O*-linked glycans of protein Ser/Thr	UDP-GlcNAc, GlcA-β1,3-Gal-β1-O-naphthalenemethanol	1ON6, 1ON8	Retaining (Yes)	10	4.0	169
GpgS (*M. tuberculosis)*	GT81	UDP-Glc	2-position of 3-phosphoglycerate	UDP-Glc, Phosphoglyceric acid	5JSX, 4DEC	Retaining (Yes)	10	3.6	153
Mgat1 (*O. cuniculus*)	GT13	UDP-GlcNAc	α-Man of tri-antennary Man_5-$GlcNAc_2$ *N*-glycan	UDP-GlcNAc	1FOA	Inverting (Yes)	7	3.4	163
UGGT (*T. dupontii*)	GT24	UDP-Glc	Glycosylation of incompletely folded glycoproteins	UDP-Glc	5H18	Retaining (Yes)	6	3.8	186
Mfng (*M. musculus*)	GT31	UDP-GlcNAc	*O*-fucose of EGF repeats	UDP	2J0B	Inverting (Yes)	17	2.4	191
TcdA (*C. difficile*)	GT44	UDP-Glc	GTPases	UDP-Glc	3SRZ	Retaining (Yes)	6	3.6	163
MpgS (*T. thermophiles*)	GT55	GDP-Man	C2-OH of glycerate-3-phosphate	GDP-Man	2WVL	Retaining (Yes)	14	3.2	157
Mnn9 (*S. cerevisiae*)	GT62	GDP-Man	α-1,6 Man residues	GDP	3ZF8	Retaining (Yes)	15	3.5	168
MgS (*R. marinus*)	GT78	GDP-Man	C2-OH of lactate, glycerate or glycolate	GDP-Man	2Y4M	Retaining (Yes)	10	3.1	162

[a] Chain B of B3GNT2:UDP-Mg^{2+}:LNnT was used to search the Dali server (13) to identify proteins with structural similarity. Numerous proteins were identified, including multiple hits for different PDB files from the same protein. The rmsd values and residues aligned are for the PDB file with highest Z-score for a given protein.
[b] Two different PDB codes indicate separate complexes with bound donor and acceptor
[c] Number of structurally equivalent residue.

Supplementary Table S3: Kinetic parameters for wild type human B3GNT2 and mutants using UDP-GlcNAc as donor and LNnT as acceptor substrate.

Enzyme form	GFP fluorescence[a]	Donor: UDP-GlcNAc				Acceptor: LNnT			
		k_{cat}	K_m	k_{cat}/K_m	$\frac{k_{cat}/K_m}{k_{cat}/K_m\,(wt)}$ [d]	k_{cat}	K_m	k_{cat}/K_m	$\frac{k_{cat}/K_m}{k_{cat}/K_m\,(wt)}$ [d]
		sec^{-1}	*μM*	*$mM^{-1}sec^{-1}$*		*sec^{-1}*	*μM*	*$mM^{-1}sec^{-1}$*	
WT (293S) [b]	518	2240 ± 120	127 ± 23	17700 ± 4140		4230 ± 594	3430 ± 910	1230 ± 500	4230 ± 594
WT (293F) [c]	1023	3700 ± 110	158 ± 15	23400 ± 2940		4056 ± 132	3300 ± 210	1230 ± 117	
K149A[c]	869	0.471 ± 0.021	284 ± 31	1.66 ± 0.255	0.000071	1.26 ± 0.3	11900 ± 3830	0.105 ± 0.059	0.00009
D245A [c]	873	0.157 ± 0.007	96.3 ± 15.2	1.63 ± 0.33	0.000070	0.548 ± 0.118	7190 ± 2320	0.076 ± 0.040	0.000062
I276A [c]	1028	49.9 ±2.3	323 ± 42	133 ± 24	0.0057	107 ± 46	13500 ± 7930	7.98 ± 8.08	0.006
H282A [c]	1451	1750 ± 100	149 ± 25	11700 ± 2610	0.50	6020 ± 730	8950 ± 1530	672 ± 197	0.55
K288A [c]	934	124.2 ± 11.4	366 ± 79	339 ± 104	0.0145	356 ± 75	14900 ± 4000	2.45 ± 0.59	0.002
Y289A [c]	998	1.96 ± 0.04	35.4 ± 4.3	55.2 ± 7.9	0.00236	5.66 ± 1.42	11800 ± 3900	0.479 ± 0.278	0.00039
Y303A [c]	203	0.505 ± 0.019	199 ± 22	2.53 ± 0.38	0.000108	0.882 ± 0.132	6300 ± 1400	0.140 ± 0.053	0.00011
D332A	791	0.106 ± 0.005	71.3 ± 13.4	1.48 ± 0.34	0.000063	0.230 ± 0.044	6840 ± 2000	0.0337 ± 0.016	0.000027
D333A [c]	532	1.5 ± 0.1	237 ± 24	6.33 ± 0.89	0.00027	3.97 ± 0.49	11000 ± 1830	0.395 ± 0.103	0.000293
F356A	1152	302 ± 17	251 ± 39	1200 ± 260	0.051	706 ± 161	10100 ± 3700	60.4 ± 38.0	0.049172

[a] The relative expression and secretion of the GFP-B3GNT2 fusion proteins in transiently transfected HEK293 cells was determined by measuring the fluorescence of the recombinant fusion proteins secreted into the media.

[b] The GFP-B3GNT2 fusion protein expressed in HEK293S (GnTI-) cells was purified by Ni^{2+}-NTA chromatography, cleaved to remove tag sequences and *N*-glycans and further purified as described for crystallization of the enzyme catalytic domain.

[c] The GFP-B3GNT2 fusion protein (and all B3GNT2 mutants) were expressed in 293-F (wild type) cells and purified by Ni^{2+}-NTA chromatography. The fusion protein tags and complex *N*-glycans were retained for the indicated wild type B3GNT2 and mutant forms of the enzyme during kinetic analyses.

[d] Values for k_{cat}/K_m for all B3GNT2 mutants were compared with k_{cat}/K_m values for wild type B3GNT2 expressed in 293-F (wild type) cells.

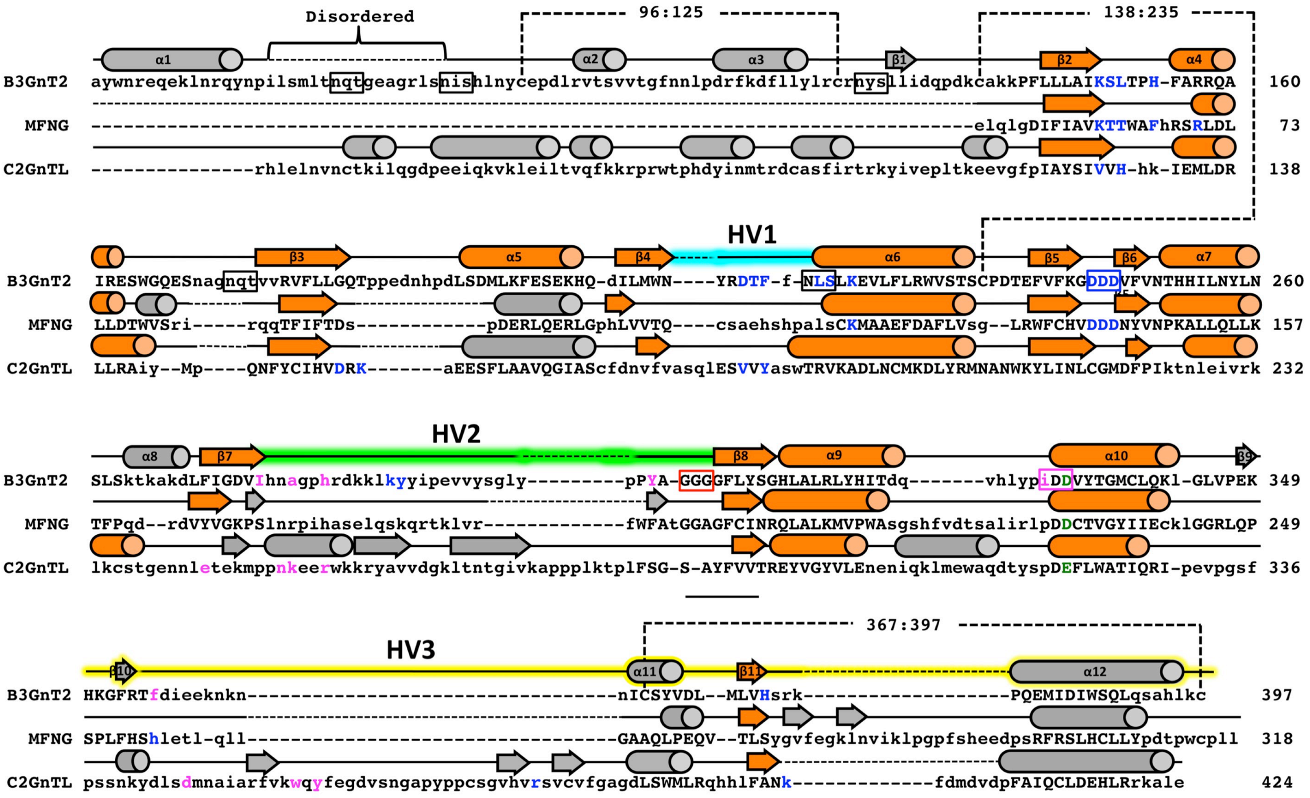

Figure S1. Sequence and structural alignment of B3GNT2 with related GTs. A structure-based sequence alignment of human B3GNT2 (residues 57-397), mouse manic fringe (Mfng, residues 50-318) and murine leukocyte core 2 GnT (Gcnt1, residues 51-424) performed using Dali (16). Residue numbers are on the right-hand side of the alignment; secondary structure is shown on top as cylinders (*α*-helices), arrows (β-sheets) and black lines (loops and turns). Structurally equivalent residues are in uppercase and non-equivalent amino acids in lower case. Secondary structure elements are numbered sequentially; those of the core GT-A fold that are conserved between the structures are colored orange. The non-conserved, inserted segments in the GT-A fold are colored grey. The three hypervariable loop insertions in the core GT-A fold (HV1 in cyan, HV2 in green and HV3 in yellow) are labeled and highlighted. *N*-glycosylation sites are identified using a black outline and disulfide bonds with dotted lines and participating residue numbers. Residues interacting with the donor (blue), acceptor (magenta) and the catalytic residue (green) are depicted using colored lettering and were obtained from the respective references for Mfng (17) and Gcnt1 (18, 19). B3GNT2 and Mfng residues belonging to the donor templates are incomplete due to the absence of the donor sugar in the respective UDP-Mg^{2+} complexes. Boxed areas indicate the residues of the *DxD* motif (blue), the '*Glycine-rich*' loop (red), and the *xED* motif (magenta).

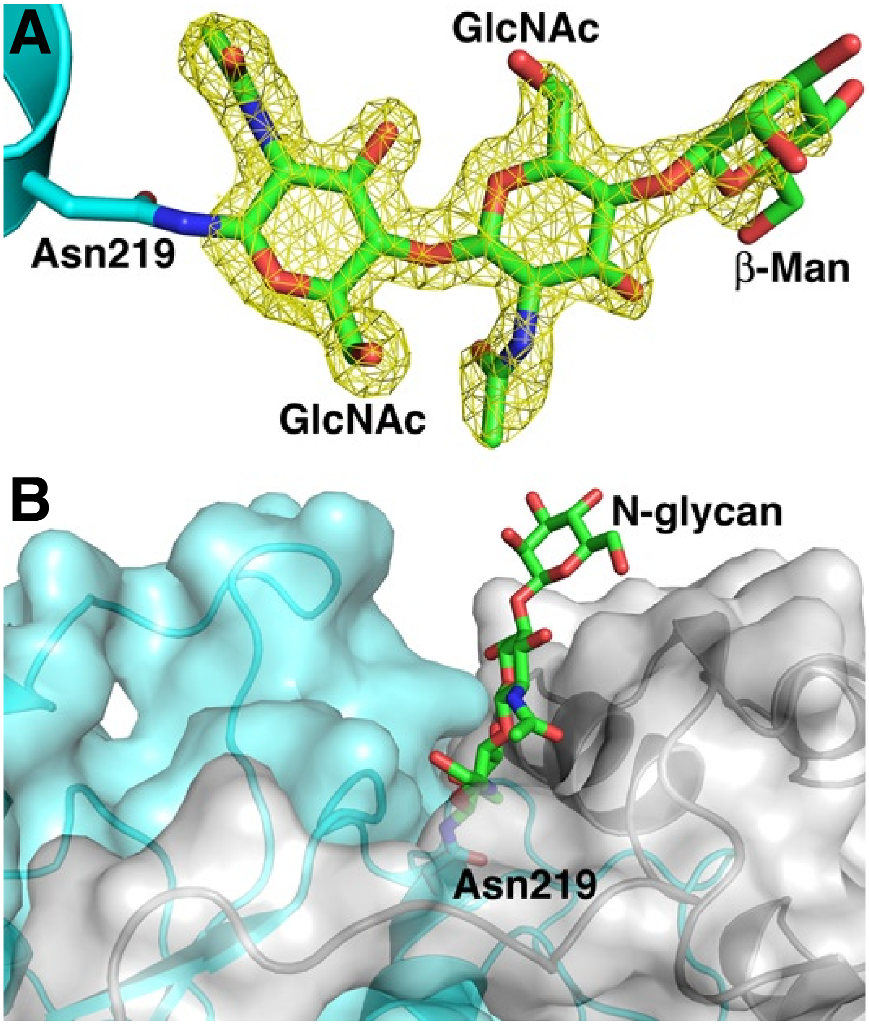

Supplementary Figure S2. The *N*-glycosylation at residue Asn219 in SeMet-B3GNT2:UDP-Mg^{2+}. (***A***) Difference density map (F_o-F_c, yellow mesh) for the *N*-glycosylation at Asn219 (green, stick mode) calculated at 1.55 Å and contoured at 3.0 σ. The map was calculated subsequent to the structure solution and an initial round of restrained refinement but prior to the modeling of the ligands. The refined coordinates of SeMet-B3GNT2:UDP-Mg^{2+} (cyan) were used for the model. (***B***) Molecular surface of the GT-A fold (cyan) and NH_2-terminal residues (52-137, pale grey) of SeMet-B3GNT2:UDP-Mg^{2+} (cartoon mode) showing the location of the *N*-glycan (green) attached to Asn219.

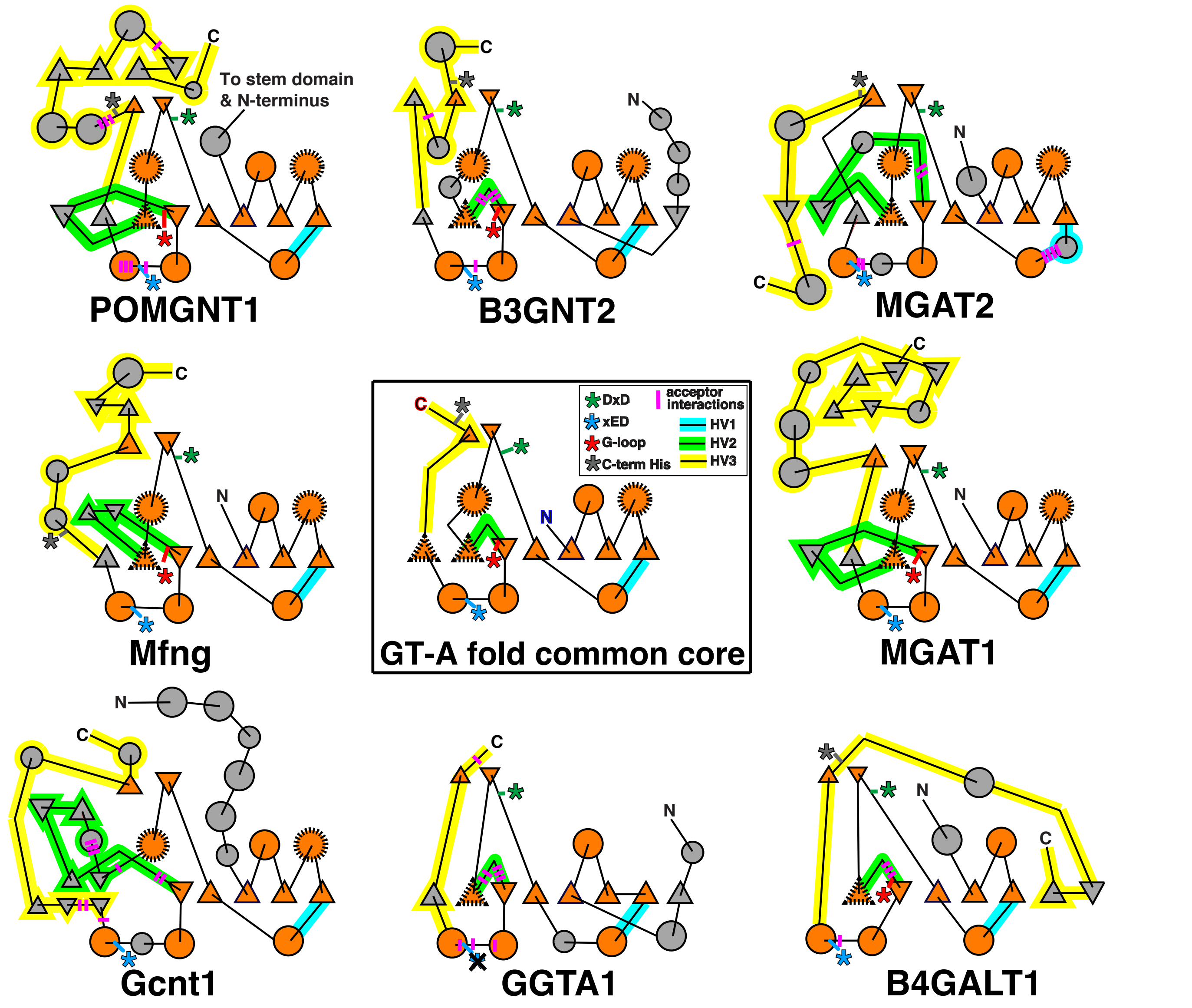

Figure S3. Comparison of the acceptor template modules in inverting and retaining GT-A fold enzymes. From the left to the right, the topology diagrams of the crystal structures of POMGNT1 (PDB code 5GGI (57)), B3GNT2, MGAT2 (PDB code 5VCS (42)), Mfng (PDB code 2J0B (30)), MGAT1 (PDB code 1FOA (39)), Gcnt1 (PDB code 2GAM (41)), GGTA1 (PDB code 5NRB (50)) and B4GALT1 (PDB code 1TW5 (47)) are shown with α-helices (circles), β-sheets (triangles) and loops (thin lines). Conserved (orange), partially conserved (circles and triangles with dashed outlines) and non-conserved (grey) structural elements of the GT-A fold are shown. The *DxD* motif in metal-dependent GTs (green asterisk), the *xED* motif containing the catalytic base (blue asterisk, except for GGTA1, a retaining enzyme that retains a carboxylate at this position (black X), but uses the donor phosphate as catalytic base)), the '*Glycine-rich*' loop (red asterisk) and the C-terminal His involved in coordinating the metal ion (grey asterisk) are indicated. Residues involved in acceptor interactions (except for Mfng and MGAT1) are depicted as dashes and colored magenta. Based on the conserved elements identified in a prior GT-A fold sequence analysis study (37) a "Progenitor GT-A fold" is depicted in the center. The acceptor template modules in inverting and retaining GT-A fold enzymes (regardless of

metal-dependence) assembled from the three hypervariable loop insertions in the core GT-A fold (HV1 in cyan, HV2 in green and HV3 in yellow) are highlighted.

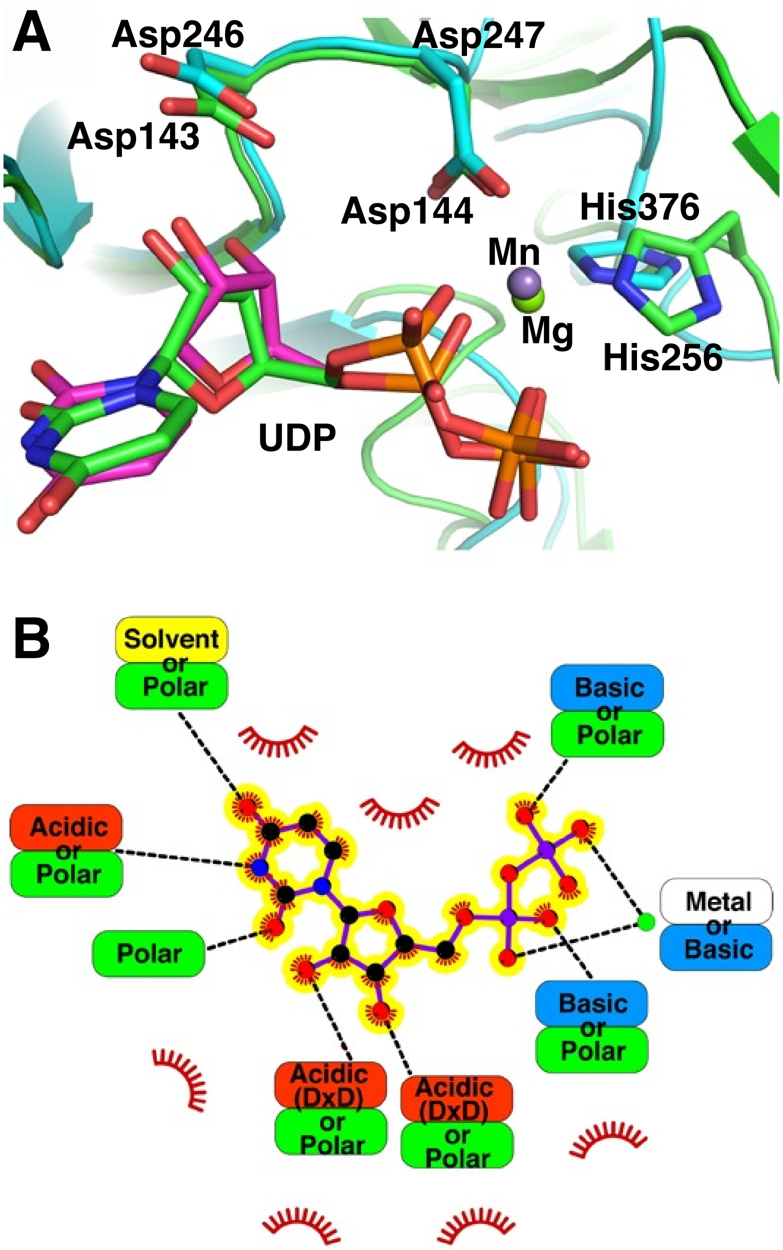

Supplementary Figure S4. Interactions of the UDP donor-analog in B3GNT2 and other GT-A fold GTs. *(**A**)* Structural alignment of SeMet-B3GNT2:UDP-Mg^{2+} (cyan) and Mfng (green) showing the replacement of His376 in B3GNT2 by a residue that originates from a different area of the Mfng structure (His256). The bound UDP (stick mode) is colored magenta (B3GNT2) or green (Mfng). The metal ions in the active site are shown as spheres. (***B***) In inverting and retaining GT-A fold glycosyltransferases, the residues that interact with the nucleotide sugar donor are not conserved at the sequence level but originate from amino acids in similar locations of the fold. The different types of interactions present are depicted as colored boxes around the UDP (stick mode, highlighted yellow). Packing interactions (red, feathered arcs), hydrogen bonds (black, dotted lines) and the metal-ion (green sphere) are shown.

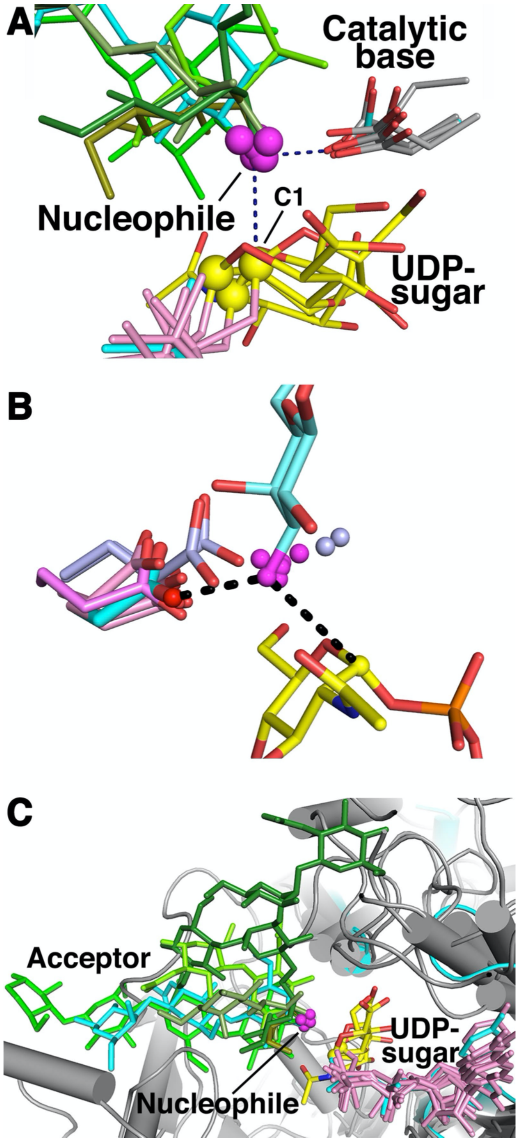

Supplementary Figure S5. Relationship of the sugar donor and acceptor nucleophile hydroxyl positions in inverting and retaining GT-A fold enzymes. (***A***) Structural alignment illustrating the position of the catalytic base (grey sticks, B3GNT2 in cyan) and the acceptor glycan nucleophile (magenta sphere) relative to the C1 atom (yellow sphere) of the UDP-sugar donor (pink with sugar residue in yellow) in metal-dependent, inverting GTs: B3GNT2, MGAT2 (42), POMGNT1 (57), B4GALT1 (47), B3GAT3 (49) and BCSA (80). The nucleotide sugar donor is bound in MGAT1 (UDP-GlcNAc), B4GALT1 (UDP-Gal) and B3GAT3 (UDP-GlcA); the other structures have bound UDP. The different glycan acceptors are depicted in shades of green. The interaction between the acceptor nucleophile hydroxyl, the catalytic base and the C1 atom of the sugar donor is depicted with black dashed lines. The alignment was constructed using the nucleotide ribosyl and diphosphate atoms and the main chain atoms of the catalytic base. (***B***) Mechanism-specific clustering of acceptor nucleophilic hydroxyls is shown with two distinct classes of nucleophile positioning. Inverting metal-dependent enzymes that employ Asp (pale pink sticks, B3GNT2 in cyan) or Glu (dark pink sticks) as catalytic base form a tight cluster for their respective acceptor nucleophilic hydroxyl (magenta spheres). Metal-dependent and metal-independent inverting enzymes that employ Glu as catalytic base (slate sticks)

form a distinctly positioned cluster for their nucleophilic hydroxyls (slate spheres). The nucleophile (magenta sphere) in the B3GNT2 acceptor glycan (cyan sticks) is shown relative to the C1 atom (yellow sphere) of the aligned donor sugar (yellow, from MGAT1:UDP-GlcNAc complex). The enzymes depicted are a representative subset consisting of those in panel (A) along with metal-independent, inverting GTs: Gcnt1 (41) and XylT1 (48) (***Supplementary Table S2***). (***C***) The structural diversity of the acceptor glycans (shades of green) relative to LNnT (cyan) in B3GNT2 (cyan). The same subset and superposition as in panel (A) are shown with the aligned nucleotide donors (pink) and donor sugars (yellow) relative to the bound UDP (cyan) in B3GNT2.

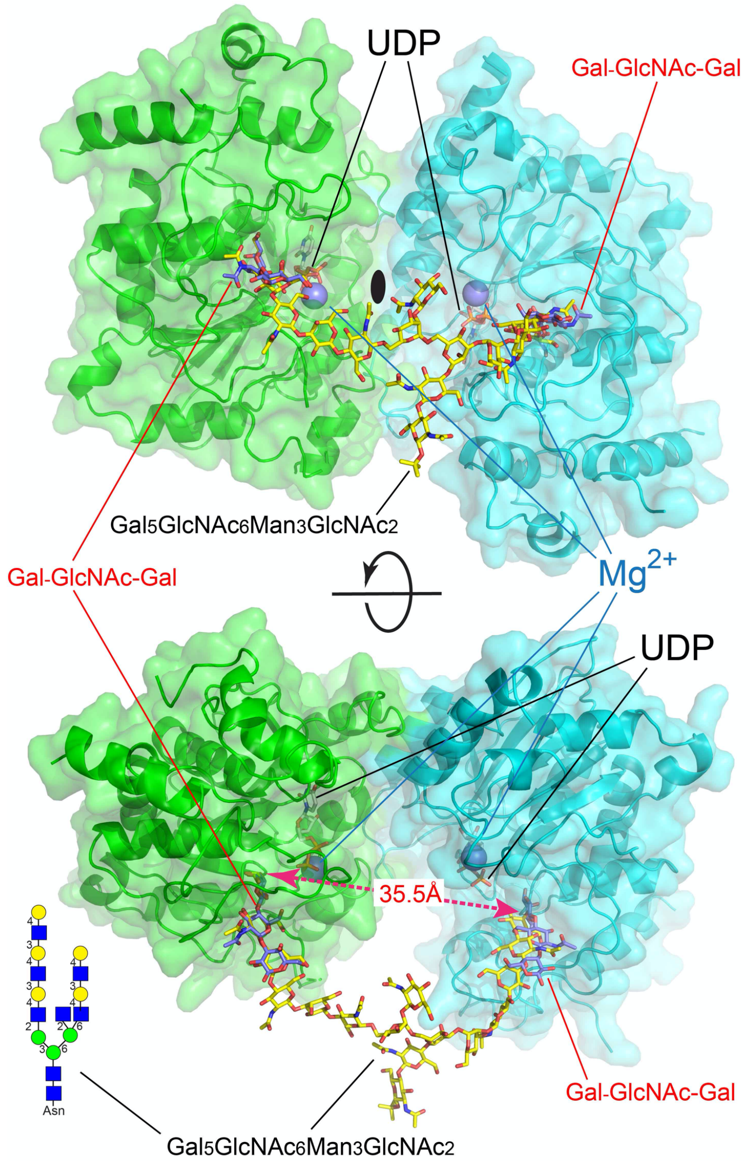

Supplementary Figure S6. Two different views of a surface and cartoon rendering of the two-fold symmetry for the B3GNT2 dimer (green and cyan chains) indicating that both active sites are on the same face of the homodimeric enzyme. (***A***) Face-on view of the B3GNT2:UDP:Mg^{2+}:LNnT dimer showing the UDP (white, sticks), Mg^{2+} (purple sphere) and the Gal and GlcNAc units of LNnT (slate blue, sticks). A multi-antennary *N*-glycan ($Gal_5GlcNAc_6Man_3GlcNAc_2$, yellow sticks) modeled into the acceptor binding site is also shown. (***B***) The edge-on view of the B3GNT2 dimer. The 35.5 Å spacing between the two active sites of the B3GNT2 dimer is indicated (red arrows) to show that only extended multi-antennary *N*-glycan structures can bridge the two active sites of the B3GNT2 homodimer.
